# Agent-based modeling for personalized prediction of an experimental immune response to immunotherapeutic antibodies

**DOI:** 10.1371/journal.pone.0324618

**Published:** 2025-06-09

**Authors:** Omri Matalon, Andrea Perissinotto, Kuti Baruch, Shai Braiman, Anat Geiger Maor, Eti Yoles, Ella Wilczynski, Uri Nevo, Avner Priel

**Affiliations:** 1 ImmunoBrain, Ltd., Rehovot, Israel; 2 Department of Biomedical Engineering, The Iby and Aladar Fleischman Faculty of Engineering, Tel Aviv University, Tel Aviv, Israel; 3 Sagol School of Neuroscience, Tel Aviv University, Tel Aviv, Israel; 4 Faculty of Engineering, Ruppin Academic Center, Emek Hefer, Israel; Rutgers: Rutgers The State University of New Jersey, UNITED STATES OF AMERICA

## Abstract

Targeting immune checkpoint pathways to evoke an immune response against tumors has revolutionized clinical oncology over the last decade. Antibodies that block the PD-1/PD-L1 pathway have demonstrated effective antitumor activity in cancer patients and are approved for treatment of several different types of cancer. However, many patients do not experience durable beneficial clinical responses. The ability to predict response to immunotherapy is a clinical need with immediate implications on the optimization of oncologic treatments. In this work we developed and tested the ability of an Agent-Based Model (ABM) to predict the ex vivo immune response of memory T cells to anti-PD-L1 blocking antibody, based on personalized immune-phenotypes. We performed mixed lymphocyte reaction (MLR) experiments on blood samples of healthy volunteers to model the dose-response kinetics of the immune response to anti-PD-L1 antibody. Additionally, immunophenotype of peripheral lymphocyte and monocyte populations was used for modeling and prediction. In silico MLR experiments were conducted using the ABM-based Cell Studio Platform, and the results of ex vivo vs. in silico experiments were compared. Our ABM accurately recapitulates MLR-derived immune responses, achieving >80% predictive accuracy. Notably, given the relatively small cohort tested, such results are typically impossible to model with methods based solely on statistical or data-driven approaches. Importantly, the use of this modeling strategy not only predicts the outcome of the immune response, but also provides insights into the exact biological parameters and related cellular mechanisms that lead to differential immune response.

## Introduction

### Immune checkpoint blockade for cancer immunotherapy

The immune system’s critical role in preventing cancer development is well-recognized. T cells are adept at identifying and eradicating aberrant cells, including those of cancerous origin, through a well-orchestrated immune action involving both innate and adaptive responses. To safeguard normal cells from unintended harm or autoimmune disorders, an array of immune checkpoints has evolved. However, cancer cells may manipulate these checkpoints to evade immune detection and elimination, undermining anti-cancer immunity. Effective anti-tumor responses necessitate the activation of T cells against tumor-specific antigens, their migration to and infiltration into the tumor site, and their local activation to destroy the cancer cells. This process must be finely balanced to ensure proper resolution, encompassing the removal of destroyed tumor cells and the restoration of normal tissue structure and function [1].

The co-inhibitory receptor, programmed cell death-1 (PD-1), expressed on immune effector cells, plays a crucial role in shielding normal tissues during immune reactions by modulating the activity of these cells. Its known ligands, programmed death ligands (PD-L1 and PD-L2), are expressed on various cells, including cancerous ones, and are recognized for their role in mediating immune escape [2]. The PD-1/PD-L1 pathway moderates the immune reaction, maintaining a balance between T cell activation and the preservation of healthy tissues, thus ensuring peripheral tolerance [3–5]. This pathway reflects an adaptive resistance mechanism used by tumor cells against the body’s own anti-tumor immunity. Overexpression of PD-L1 is observed in tumor cells and in the non-transformed cells within the tumor microenvironment [6]. When PD-L1 on tumor cells binds to PD-1 receptors on activated T cells, it leads to the suppression of these cytotoxic cells, thereby impairing immunosurveillance.

The evolving concept in contemporary immunotherapy focuses on reactivating dormant immune responses by targeting the inhibitors that maintain T-cell inactivity and immune tolerance [7]. The PD-1/PD-L1 axis and the resulting immune tolerance to cancer cells can be counteracted by checkpoint blockade antibodies directed against PD-1 or PD-L1 molecules. Therapies using monoclonal antibodies against PD-1 and PD-L1 have become standard immunotherapeutic treatments for a variety of cancers.

PD-1/PD-L1 blockade was also suggested as a potential therapy in the context of neurodegenerative diseases. Specifically, administration of either anti-PD-1 or anti-PD-L1 antibody was shown to reduce brain pathology and improve cognitive performance in various mouse models of Alzheimer’s disease [8–12]. In neurodegenerative diseases, the effect of blocking anti-PD-1/PD-L1 on mitigating brain pathology was associated with evoking an immune response in the periphery (outside the brain) which induced recruitment of specialized immune cells to the brain’s territory. Once inside the brain, these immune cells were shown to mediate reduction in the brain inflammatory profile and removal of toxic components, leading to increased survival of neurons and improved cognitive performance. This approach is currently being studied in an ongoing Phase 1 clinical trial (NCT05551741) in persons with Alzheimer’s disease for intravenous IBC-Ab002, a novel fully human anti-PD-L1 antibody [13].

Despite the high efficacy of immunotherapies in treatment of cancer and other conditions, a large group of cancer patients fails to respond to immune checkpoint inhibitors (ICI). This lack of response may be caused by various factors, such as the development of resistance mechanisms, T cell dysfunction, and the low availability of PD-1 or PD-L1 as targets, leading to treatment discontinuation [14,15]. Considering the high costs and low life expectancy of non-responding patients, there is a need to select potential responders before therapy.

In this work we address this challenge of identifying responders vs. non-responders by using Agent-Based Modeling (ABM) to simulate the ex vivo response of autologous immune cells from different donors to anti-PD-L1 antibody.

### Agent-Based Modeling

ABM is a powerful technique to simulate and explore phenomena that include a large number of active components represented by agents. In the ABM framework the agents operate in a simulated environment, where they both influence their surroundings and are influenced by the simulated environment. The agents can also perform actions autonomously, based on rules or state machine(s), with regards to their interaction with other agents and with the environment. These actions represent the behavior in the real system [16]. Modeling of a multi-scale spatiotemporal system is a complex computational challenge due to the high number of sub-processes involved, each with its own features. As the complexity of the simulated system increases in size and in the agents’ capabilities, the outcome of the simulation may reveal unexpected (emergent) results that are otherwise very hard to obtain, e.g., by pure mathematical modeling [17]. ABM requires mainly local knowledge regarding the mechanisms (rules) that govern the behavior of each type of agent and the environment. Global behavior emerges from these local agent-agent interactions as well as the interactions with the environment.

Cell Studio [18,19] is a unique platform for modeling complex biological systems. It provides an advanced environment, specifically designed for non-coding researchers, including a visual interface, allowing modeling of biological, biophysical, bioinformatics and chemical data. The platform uses high performance computing (HPC) and parallel computing. In particular, Cell Studio specializes in modeling immunological response at the cellular level. The current version of the system is not yet available for public use.

Cell Studio’s main goal in this work is to realistically describe and model the immunological response as a multi-scale, hierarchical phenomenon that operates at the molecular and cellular levels. Other applications involve modeling the immune response at the tissue and organ levels. The platform, adopting the ABM paradigm, proposes the cells as the native agents whose interactions with proteins, molecules, medium and other cells, define the main features of the immunological scenario. The types of interactions and rules necessary to describe a particular biological process are selected by the user using a setup-GUI. To facilitate the modeling process, we use a state-machine description of each cell type to control its dynamic behavior (as an approximation for the intracellular processes), in line with the growing number of publicly available pathways and network databases. For model parameterization, experimental data describing the kinetics of the immune response and anti-PD-L1 antibody effects was used.

In this work, we aimed to test the power of Cell Studio in predicting an immune response to immunotherapy. Specifically, Cell Studio was tested in a series of ex vivo autologous Mixed Lymphocyte Reaction (MLR) experiments designed to assess the immunological response of cells, derived from human peripheral blood mononuclear cells (PBMCs), when a specific anti-PD-L1 antibody is added. This antibody is designed to block the PD-1/PD-L1 interaction, thereby potentially activating the corresponding T cells. Below we describe the platform and its value in predicting the personalized response (or lack of response) of individuals’ T cells in the ex vivo MLR assay.

## Materials and methods

### Experimental approach for agent-based modeling

The modeling was based on analysis of human blood samples from healthy volunteers.

Samples underwent immune profiling to monitor differences in the immune phenotype of different subjects. Samples were then used in MLR experiments in the presence of different concentrations of anti-PD-L1 antibodies. The outcome of the MLR experiments and immune profiles served as the observations for ABM development and for personalized immune response predictions.

### Ethics statement

The study was conducted in compliance with the ethical standards outlined by Tel Aviv University’s Academic Secretariat and the University Ethics Committee. Institutional approval was obtained from the Tel Aviv University Ethics Committee (prof. M. Lahav), confirming institutional review and endorsement.

Blood samples from anonymous healthy donors were provided by Magen David Adom in Israel (MDA, mdais.org), the Israeli National Blood Bank. MDA was solely responsible for donor interactions and obtained informed consent from all participants prior to sample collection. MDA maintained complete donor anonymity, with no direct contact between the research team and the donors. Blood samples were accessed for research purposes between May 2019 and March 2020.

### Human PBMC isolation and cryopreservation

Buffy coats from venous blood of normal healthy volunteers were obtained from the Blood bank of Israel (Ramat Gan, Israel), collected according to Israeli ministry of health guidelines. PBMCs were isolated using Lymphoprep (Stemcell; cat. #07851) by standard density-gradient centrifugation. The amount of PBMCs per sample was assessed by live cell count with Trypan blue. PBMC samples were resuspended with serum-free freezing medium (Biological Industries; catalog# 05-065-1A) at a concentration of 30x10^6^ cells/vial, and immediately cryopreserved in −80°C using a CryoCooler Freezing Container (containing isopropyl alcohol). 24 hours later, samples were transferred to liquid nitrogen.

### Autologous mixed lymphocyte reaction (MLR)

The autologous MLR consists of incubation of memory CD45RO^+^ T cells with CD14^+^ monocytes, isolated from blood samples of the same individual, and soluble anti-CD3 antibody to drive T cell activation. The monocytes, which are professional antigen presenting cells (APC), express co-stimulatory receptors and the inhibitory molecule PD-L1, thereby regulating the activation of T cells. In this assay, the T cells and monocytes are co-cultured for several days in the presence of increasing concentration of the anti-PD-L1 antibody, or with IgG isotype control. Intensity of T cell activation is measured in terms of cytokine secretion (i.e., interferon-gamma (IFNγ)) and expression of the T cell surface activation marker, PD-1, which is upregulated over stimulated T cells. Monocyte and memory T cells were isolated from thawed PBMCs by magnetic separation using human CD14 microbeads (Miltenyi Biotech; cat. #130-050-201), and by magnetic separation using human CD45RO microbeads (Miltenyi Biotech; cat. #130-046-001), respectively. The mixed cells were plated in 200μl medium (RPMI with 10% heat-inactivated fetal bovine serum (hiFBS)) in a 96 well flat bottom plate at 125x10^3^ monocyte and 75x10^3^ memory CD45RO T cells per well. Cells were incubated (37⁰C, 5% CO2) for 1−5 days, with or without the activator soluble Leaf^TM^ anti-human CD3 (Biolegend; cat.#300414) and the tested anti-PD-L1 Monoclonal antibodies (mAbs). Each experimental treatment dose (serial 10−5 fold titration of the antibodies, ranging 10^−13^ to 10^−7^ M) were performed in triplicates.

The anti-PD-L1 concentration range of 10^-13^ to 10^-7^ M (0.000015–15 µg/ml) was selected to capture both subthreshold and saturating concentrations, ensuring the generation of a robust dose–response curve, based on previous reported in vitro ranges from prior immunotherapy assays and standard monoclonal antibody behavior. The higher end of this range (10^-7^ M) overlaps with nanomolar plasma/tissue levels that can be transiently reached by therapeutic antibodies in vivo. The lower end (10^-13^ M) helps detect any early or minimal cellular responses that might occur at near-threshold antibody concentrations.

Following the incubation period, supernatants were collected and IFNγ was measured by ELISA (Biolegend; Human IFNγ ELISA MAX™ Deluxe, cat. #430105) according to manufacturer’s instructions. In addition, immune cells were collected for flow cytometry analysis to measure the expression of PD-1 on T cells and other cell surface markers (see supplementary material in S1 Text and S1 Fig).

Multiple incubation times (24, 48, 72, and 96 hours) were evaluated to capture the kinetic profile of T cell activation and cytokine release. Preliminary assays showed that some activation markers, such as PD-1 upregulation and IFNγ secretion, were not robustly elevated at 24 hours; hence, longer incubation durations were essential for detecting the full immunological response.

### Agent Based Modeling (ABM)

**Fig 1:**
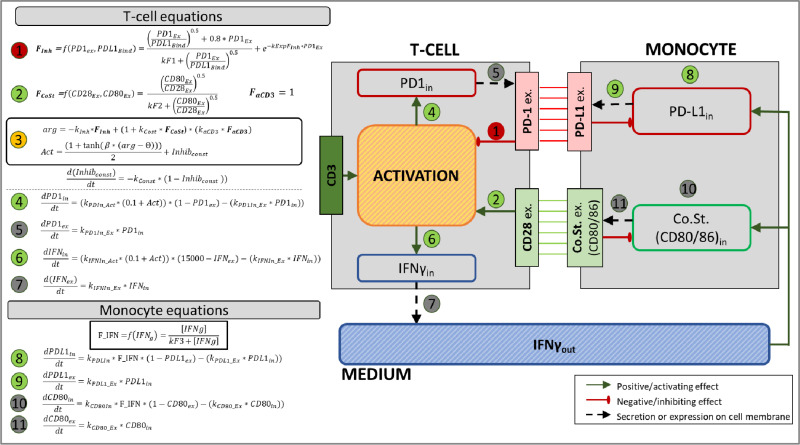
Summary of MLR regulatory network and corresponding equations defining the state machine. The left-hand side of the figure describes the equations governing each signal in the model depicted on the right-hand side. The equations’ details are elaborated in the material and methods section, as well as in the supporting information; the considerations and assumptions underlying the equations are discussed in detail throughout the manuscript. The model diagram depicts the interaction between the two cell types, T cell and Monocyte, as well as their interaction with the environment (denoted as ‘Medium’). The two cells interact via receptors expressed over the T cells (PD-1, CD28) and Monocytes (PD-L1, CD80/86), respectively. Equations 1−11 can be divided into the following groups, governing key processes that determine the T cell activation state: (Equations 1–3) Define T cell activation state by integrating upstream regulatory signals. Equation 1 models the inhibitory effect of PD-1/PD-L1 binding, while Equation 2 describes the co-stimulatory effect of CD28/CD80 binding. Equation 3 combines these inputs, alongside CD3 signaling. (Equations 4–7) Govern dynamic T cell responses through differential equations, including PD-1 intracellular expression (Equation 4), PD-1 extracellular dynamics (Equation 5), IFNγ secretion (Equation 6), and its diffusion into the medium (Equation 7). The time-dependent variables of these equations influence the regulatory terms in Equations 1–3 to determine the activation state of the T cell. (Equations 8–11). Model monocyte responses, including PD-L1 expression upregulated by IFNγ (Equations 8–9) and CD80/86 dynamics (Equations 10–11) influenced by the cytokine environment. The ‘Activation’ block in the T cell represents the overall state of the cell; the value of this internal variable was not measured experimentally, therefore its effect on the cell behavior can be evaluated only indirectly, i.e., by its effect on the measured variables of activation biomarkers. The T cell activation level is upregulated by the CD3 and CD28 and inhibited by the expressed PD-1 level. The activation level regulates the expression level of PD-1 and IFNγ. The Monocyte dynamics is represented by the level of the PD-L1 and the co-stimulatory internal molecules and receptors CD80/86. The monocyte receptor expression also depends on the level of IFNγ in the medium released by the T cells.

As described earlier, ABM requires local knowledge regarding the mechanistic and other rules that govern the behavior of each type of agent and the environment. In our case, we focused on two types of leukocyte cells, T cells and Monocytes. In the following we provide the concepts behind the mathematical modeling of these cells, as well as of the environment. Full description of the model is given in the Supporting Information (supplementary Material and Methods section in S1 Text). A high-level description of the ABM framework, including the categorization of equations and their interactions, is provided in Fig 1. Cell Studio code used in this study is available from GitHub at https://github.com/cellstudiotau2019/CellStudio_Run.

### Leukocytes detailed modeling

The leukocytes behavior is defined through the state machine depicted in Fig 1. It is assumed that upon physical connection, the T cell first scans the other cell and the result of this process is either disengagement, or the initiation of immunological synapse. This model is implemented in Cell Studio and evaluated for each agent (cell) during the simulation. The full mathematical description of the model is provided in the Supporting Material and Methods; key equations are also given below for clarity.

#### T cell.

In our simplified model of a T cell, it expresses on its membrane the following three types of relevant receptors/co-receptors: CD3, CD28 and PD-1. The CD3 can bind to its antibody, anti-CD3, which is applied to the solution at the beginning of the experiment. The stimulatory effect of anti-CD3 binding is defined by the variable FaCD3 (see Equation (Eq) S1 in S1 Text). The other two receptors can bind to their ligand expressed on the monocyte: the CD28 can bind to CD80/ CD86, while the PD-1 binds to PD-L1 (which has affinity also to the antibody anti-PD-L1). These two processes are defined by the variables FCoSt and FInh (see Eqs S2 and S3 in S1 Text), respectively. The expression of the receptors on the T cell and their occupancy level define the behavior and, thus, the activation of the T cell. Unlike the other variables, which correspond to actual measured quantities, the ‘Activation’ is a hidden variable (not directly measured) and describes collectively the activation state of the T cell, as a function of the above variables.

The combined contribution is described in the state-machine of the overall activation below (and also described in Eqs S4 and S5 in S1 Text):


$$Act=\frac{\left(1+\tanh\left(\beta \cdot \left(A_{coeff}-\theta \right)\right)\right)}{2}+Inhib_{general}$$

where,


$$A_{coeff}=-k_{Inh}\text{*}F_{Inh}+(1+k_{Cost}*F_{CoSt}\text{)}*(k_{aCD3}*F_{aCD3})$$

and


$$\frac{d(Inhib_{general})}{dt}=-k_{general}*(1-Inhib_{general\text{\textasciitilde{}}}))$$

where $F_{Inh}$, $F_{CoSt}$, $F_{aCD3}$ are the functions of the three processes described above, and $k_{Inh}$, $k_{Cost}$, $k_{aCD3}$ are the weights given to these three processes. The main reason for using a hidden variable is the difficulty to directly connect the internal (production) level of PD-1 (and the IFNγ) to the membrane expression level; the internal level was not measured. The expression dynamics of PD-1 and IFNγ is described in Eqs S6 and S7 in S1 Text. Note that since the expression of PD-1 is given in percentage, and not in actual receptors counting, the activation defined above was included as a separate function to impose its range to [0, 1]. Similarly, the variable $Inhib_{general}$ takes into account other inhibitory mechanisms that are not measured directly, as a collective mechanism.

#### Monocyte.

The model of monocyte is different from the T cell model due to the absence of an activation variable. The two receptors chosen to represent the monocyte activity are PD-L1 and CD80, and their expression depends on the concentration of IFNγ (Eq S8 in S1 Text) sensed by the monocyte. The expression dynamics of PD-L1 and CD80 is defined by a function of the IFNγ concentration that normalizes its value. The production and expression of PD-L1 and CD80 follows the same model as for PD-1 in T-Cell: initially the receptors are produced in the cell and after some time (stochastic variable) they are expressed on the surface. The equations describing PD-L1 and CD80 dynamics are detailed in the supporting information (Eqs S9 and S10 in S1 Text).

### Algorithmic and complexity considerations

Since ABM is a highly intensive computational framework, the implementation of certain modules in Cell Studio were optimized. The following list describes some of the endeavors taken.

1. Repeated actions: There are several types of actions that are repeated in various time scales, many of which represent physical processes such as diffusion of molecules in the medium. For this reason, these processes are evaluated with different frequencies whose value is defined based on the rate of the process (faster processes are evaluated more often). By controlling the update rate per action, we can optimize the overall time required by these processes.
2. Motion: The motion of a cell/agent is not continuously evaluated but only when the motion is expected to generate a relevant change in the state of the cell and/or its neighbors (e.g., collision). For each cell movement in the simulation, an “interaction map/graph” is evaluated, containing information on the agents that are expected to interact, and on the expected time and location. This reduces considerably the motion-related computation, by allowing frequent updates only in the time window when an interaction is expected.
3. Environment update: The total volume simulated is divided into a 3D grid of voxels with a defined spatial resolution (50μm). The choice of the grid step can optimize computation time, while keeping the sensitivity necessary to accurately model the experiment. For the same reasons, updating the environment, as for the modeling of diffusion of secreted molecules, is done with varying temporal and spatial steps.
4. Parallel Infrastructure: Using parallel computing, it is possible to run multiple simulations simultaneously, as separate processes/ instances. This feature allows the modeler to repeat a certain simulation multiple times, possibly with different runtime parameters, including running multiple instances in parallel, on separate machines/ processors. This allows the modeler to explore a parameter space and obtain results at a much faster pace.

### Model validation

To examine and to quantitatively validate the performance of Cell Studio, in silico experiments were conducted, and their results were compared to those of ex vivo experiments, using blood samples of healthy donors. Each run of Cell Studio with a particular set of parameters (donor immunophenotype data, antibody concentration etc.) simulated a 4–5-days experiment. In parallel, ex vivo experiments were conducted to obtain the dose response curve for various variables, such as the level of receptors expression and the concentration of cytokines secreted.

The model simulated the in silico level of immune response according to the following input data: (1) immunophenotype baseline expression levels of PD-1 on T cells; (2) immunophenotype baseline expression of PD-L1 on monocytes; and (3) concentrations of the anti-PD-L1 antibody in the medium. The predictions resulting from the in silico immune response are given in the results section.

## Results

### Characterizing response to anti-PD-L1 blockade via immune activation profiles

**Fig 2:**
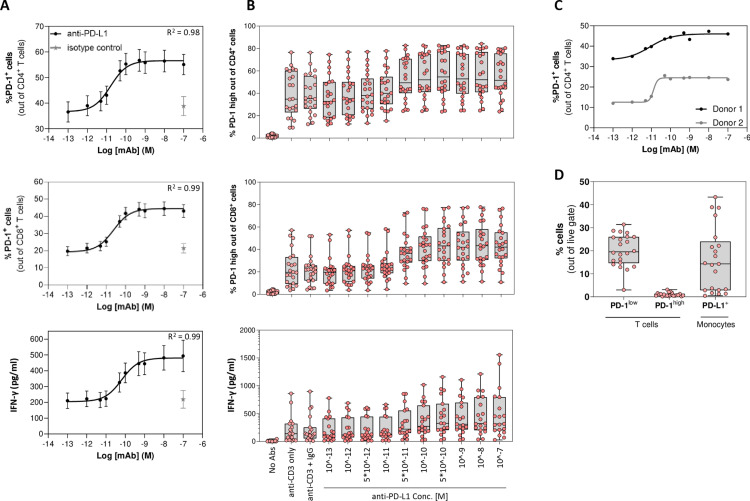
anti-PD-L1 dose effect on T cells activation in MLR experiments. (A) Antibody dose effect on T cells activation was tested using an autologous MLR setup and serial dilutions of anti-PD-L1 antibody (ranging 10^-13^-10^-7^ M), following 3−5 days of incubation. Graphs show sigmoidal curves of %PD-1^+^ cells out of the CD4 T cells (top panel; N = 22; R^2^ = 0.98), %PD-1^+^ CD8 T cells (middle panel; N = 22; R^2^ = 0.99), or IFNγ levels in the medium (bottom panel; N = 19; R^2^ = 0.99). Data for each concentration is given as mean ± s.e.m. (B) Differences in activation intensity of T cells from different donors, in response to anti-PD-L1 treatment: antibody dose effect on T cell activation was tested using an autologous MLR setup and serial dilutions of the anti-PD-L1 antibody (ranging 10^-13^-10^-7^ M) following 3−5 days of incubation. Shown are box plots and individual values per donor of %PD-1^+^ cells out of the CD4 T cells (top panel; N = 22), %PD-1^+^ CD8 T cells (middle panel; N = 22), or IFNγ levels in the medium (bottom panel; N = 19). Cells were either non-activated (No Abs), activated with anti-CD3 only (with or without isotype IgG control), or activated with anti-CD3 plus the indicated concentration of anti-PD-L1 antibody. Data are shown as box plots indicating the median and 25th and 75th percentiles, with individual data points overlaid. One-way ANOVA across the indicated anti-PD-L1 concentration groups demonstrated a significant dose effect on PD-1 expression for CD4^+^ and CD8^+^ T cells (p < 0.0001) and on IFNγ levels (p = 0.0026). (C) Anti-PD-L1 dose effect on T cell activation of two distinct donors (Donor 1 – min values:33.3%, max values: 46%, EC50: 8.5*10^-12^M; Donor 2 – min values:12.5%, max values: 24.5%, EC50: 1.3*10^-11^M). (D) Basic immunophenotyping of PD-1 expressing memory T cells and PD-L1 expressing monocytes from donors used in MLR experiments. Isolated CD45RO^+^ cells (memory T cells) and CD14^+^ cells (monocytes) were stained for PD-1 or PD-L1, respectively, to determine cell percentages at baseline (before MLR experiments). N = 22; Data are shown as box plots indicating the median and 25th and 75th percentiles, with individual data points overlaid.

The effect of anti-PD-L1 antibody on the intensity of T cell activation in MLR experiments was used to identify profiles of responding vs. non-responding healthy volunteers. T cell activation is measured in terms of IFNγ secretion and PD-1 expression on the T cell surface, which is upregulated following cell stimulation. Results demonstrate a clear dose response with all activation readouts as a function of anti-PD-L1 concentration, ranging from 10^-13^M to 10^-7^M (0.000015–15 µg/ml) (Fig 2A). Accordingly, one-way ANOVA across the tested anti-PD-L1 concentrations demonstrated a significant effect on the percentage of CD4^+^ and CD8^+^ T cells expressing PD-1 (p < 0.0001) and IFNγ secretion (p = 0.0026).

Importantly, however, although anti-PD-L1 exposure effected the majority of blood samples, the magnitude of T cell activation and/or treatment EC50 (half maximal effective concentration) differed between donors, resulting in a distribution of activation intensities, as measured by the expression of PD-1 on CD4 and CD8 T cells or IFNγ secretion (Fig 2B). An example for this phenomenon is demonstrated by differences in %PD-1 expressing CD4^+^ T cells of two distinct donors in response to anti-PD-L1 treatment (Fig 2C). These results emphasize the need for establishing a model for predicting the effect of anti-PD-L1 treatment on the immune response, potentially via personal immunophenotype readouts.

Results of baseline immunophenotype measurement of the assay, examined before the MLR experiments, are also shown (Fig 2D), and include frequencies of PD-1 expressing memory T cells and PD-L1 expressing monocytes. Similar to the MLR data, a range of baseline PD-1 and PD-L1 expression levels was observed.

### Analyzing immune response kinetics for Cell Studio development

Cell Studio simulates immunological activities using an ABM approach that depends on the kinetics of biological processes. Thus, the kinetics of T cell activation, and additional biological events, throughout the period of MLR incubation were analyzed. To this end, multiple kinetic MLR experiments were conducted to measure T cell activation at 24h, 48h, 72h, and 96 hours post incubation. Specifically, the percentage of PD-1 expressing memory CD4 and CD8 T cells and IFNγ secretion were measured.

Dose-dependent effect on T cell activation was examined in a range of anti-PD-L1 antibody concentrations. As controls, cells were activated with anti-CD3 alone (to mimic non-specific immune activation), anti-CD3 plus IgG isotype control, or were left nonactivated (without anti-CD3 and anti-PD-L1 antibodies).

**Fig 3:**
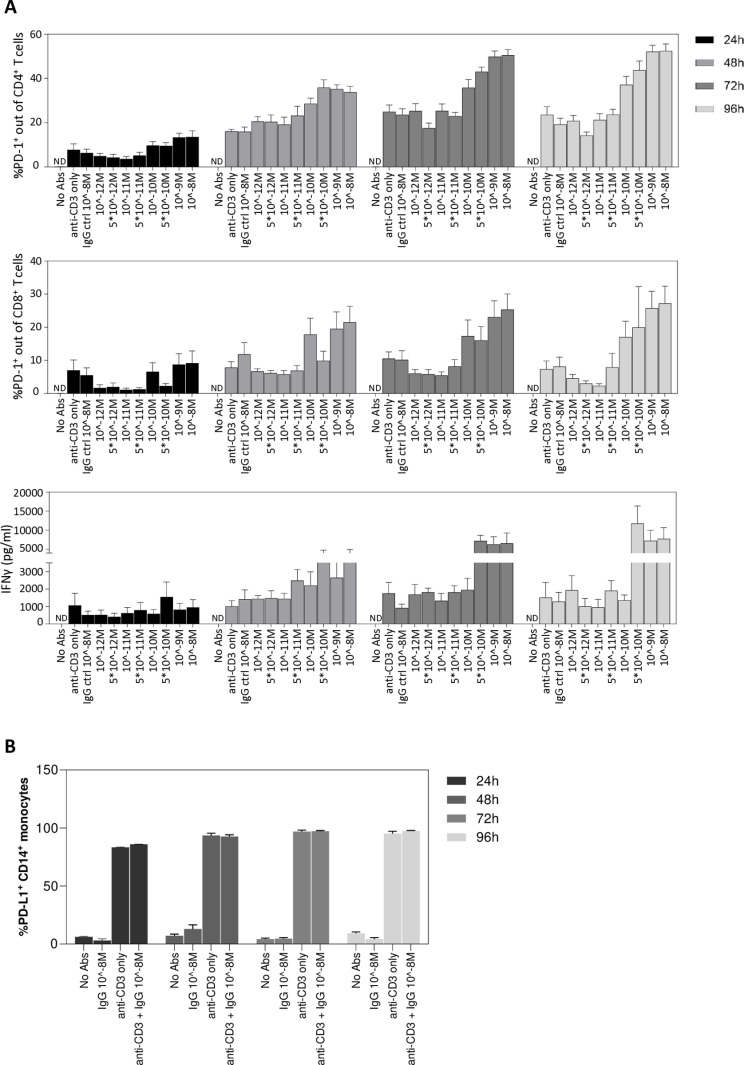
Kinetics of T cell and monocyte activation throughout 4 days of MLR experiments. **(A)** MLR experiments of 24h, 48h, 72h and 96h were conducted and %PD-1 expressing memory CD4 T cells (top panel, N = 15 donors), %PD-1 expressing CD8 memory T cells (middle panel, N = 15 donors), and IFNγ levels (bottom panel, N = 8) were determined using flow cytometry or ELISA. Data is shown as mean ± s.e.m. One-way ANOVA analysis across time points (24h, 48h, 72h, 96h) revealed p < 0.0001 for and %PD-1^+^ memory CD4 T cells, p = 0.03 for %PD-1^+^ memory CD8 T cells, and p = 0.046 for IFNγ levels. ND, not detected. **(B)** MLR experiments of 24h, 48h, 72h and 96h were conducted and %PD-L1 expressing CD14^+^ monocytes was analyzed by flow cytometry. Analysed samples included non-activated cells (No Abs and IgG 10^-8^M controls) and activated cells with anti-CD3 or with anti-CD3 plus 10^-8^M IgG isotype control. N = 3, Data is shown as mean ± s.e.m.

In terms of PD-1 upregulation over CD4 and CD8 T cells, a dose response was detected at all time-points (Fig 3A, top and middle panels), indicating a gradual increase in T cell activation over time. At 24 hours, ~ 6% increase in PD-1 expressing cells was observed in samples treated with 10^-12^M anti-PD-L1, relative to non-activated controls (~1%). Further increase was detected following treatment with higher anti-PD-L1 concentrations, demonstrating a dose dependent effect on T cells activation. PD-1 expression levels peaked at ~72 hours, and in some samples started to drop following 96−120 hours (120h data not shown). One-way ANOVA analysis across the four time points (24 h, 48 h, 72 h, 96 h) indicated a significant effect of time on PD-1 expression in both CD4 (p < 0.0001) and CD8 (p = 0.03) T cells, further supporting the kinetic changes observed in T-cell activation.

Similar trend was observed in terms of the functional output of IFNγ secretion to the medium. IFNγ was secreted following cellular activation, and its levels increased upon treatment with anti-PD-L1 in a dose dependent manner. IFNγ level peaked at ~72–96 hours (Fig 3A, bottom panel), and started to drop following 96–120 hours (120h data not shown). Consistently, one-way ANOVA revealed a significant time effect on IFNγ levels (p = 0.046), highlighting the observed temporal pattern of cytokine production.

Since PD-L1 is upregulated on immune cells in response to IFNγ, the kinetics of PD-L1 expression on monocytes was also examined. Non-activated samples (without anti-CD3 antibody or with IgG isotype control) and samples activated with anti-CD3 alone were analysed. Samples that were activated in the presence of anti-PD-L1 were not analysed since the therapeutic antibody competes with the flow cytometry antibody, used for PD-L1 staining.

In non-activated samples less than 10% of cells were PD-L1 positive. However, in activated samples, this percentage rose to approximately 90%, as early as 24 hours following incubation, and remained stable until the end of the experiments (96 hours) (Fig 3B). These results suggest that PD-L1 acquisition by monocytes occurs at an early stage of the MLR. This is essential for modeling the outcome of in silico runs that are highly dependent on the ability of PD-L1 expressing monocytes to suppress the activation of T cells via the PD-1/PD-L1 pathway.

The kinetics of monocyte death throughout the MLR was also examined (for details, see Supporting Results section and S2 Fig.

The results of the kinetic experiments were used to establish the temporal rules in Cell Studio that govern the process of T cell activation over time and with dependence of the concentration of anti-PD-L1 antibody.

### Assessing the effects of IFNγ on monocyte viability and cellular activation

Monocytes are professional antigen presenting cells (APCs), responsible for processing and displaying antigens to T cells via major histocompatibility complex (MHC) molecules on their cell surfaces. Following recognition of specific antigen, the T cells are being activated, and, subsequently, secret IFNγ to the surrounding. APCs can be activated by the secreted IFNγ, which results in expression of MHC class II and co-stimulatory molecules, such as CD80/86 (B7 complex) [20–23]. In addition, co-inhibitory molecules, such as PD-L1, are also over-expressed to ensure timely termination of the immune response [24]. This activation process renders monocytes more efficient APCs, which support robust, yet regulated, T cell dependent immunity. At the end of the process, this activation can also lead to monocyte death [25].

**Fig 4:**
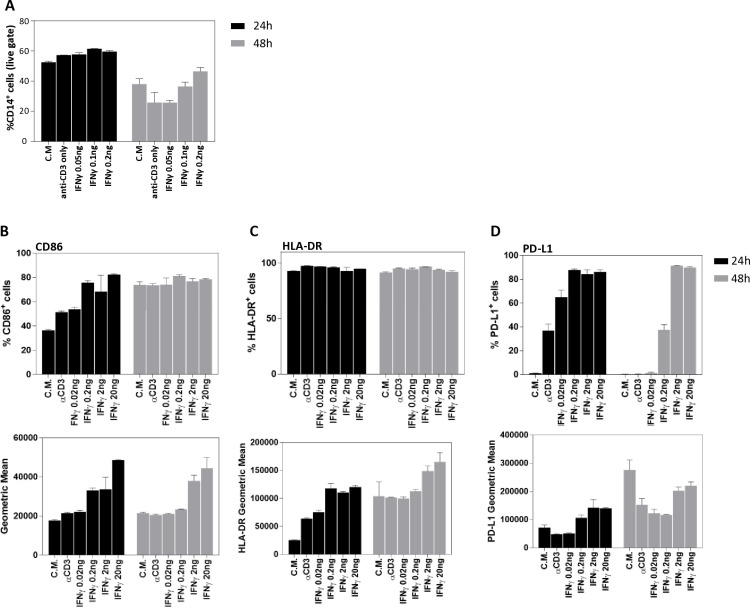
IFNγ effect on monocyte viability and activation. **(A)** Monocytes (CD14^+^ cells) were isolated from PBMCs of healthy donors (N = 3) and incubated in 0.2 ml complete medium (CM) and in the presence of 0.05ng, 0.1ng or 0.2ng rhIFNγ (concentration range 0.25-1 ng/ml) for 24 and 48 hours. As controls, cells were also incubated without rhIFNγ or with anti-CD3 antibody. The percentage of CD14^+^, out of live-gate, was determined by flow cytometry. Data is shown as mean ± s.e.m. **(B-D)** IFNγ effect on monocyte activation was determined by the expression level of stimulatory and inhibitory molecules. rhIFNγ (0.02ng-20ng) was added to isolated CD14 positive cells incubated in 0.2 ml complete medium (concentration range of 0.1-100ng/ml) for 24 and 48h. Top panels: percent of cells expressing **(B)** CD86, **(C)** HLA-DR or **(D)** PD-L1. Lower panel: geometric mean of **(B)** CD86, **(C)** HLA-DR or **(D)** PD-L1 on monocytes. N = 3 donors. Data is shown as mean ± s.e.m.

All the aforementioned events are crucial for accurate ABM of an MLR setting, and thus, the effect of secreted IFNγ on monocyte viability and activation was examined. To determine the direct effect of IFNγ on monocytes, and to prevent effects originating from T-cell-monocyte interactions, recombinant human IFNγ (rhIFNγ) was used. Monocytes were isolated from PBMCs of healthy donors and incubated in complete medium for 24 and 48 hours, in the presence of a range of rhIFNγ concentrations (0.25–1 ng/ml). As negative controls, monocytes were incubated in the absence of rhIFNγ, or with anti-CD3 antibody. Flow cytometry analysis demonstrated no significant alterations in CD14^+^ cell percent following 24 hours, as compared to the controls (Fig 4A). At 48 hours, monocyte percentage were reduced in all samples, yet no significant reduction was detected in samples incubated with rhIFNγ, relative to the negative controls (Fig 4A). These results suggest that IFNγ does not directly results in monocytes death in the MLR settings.

The effect of IFNγ on monocyte activation was also examined, using similar settings. Isolated monocytes were incubated with a range of rhIFNγ concentrations for 24 and 48 hours and the expression of co-stimulatory and inhibitory molecules were determined by flow cytometry. Co-stimulatory receptors included CD86, which serves as a ligand for the T cell co-stimulatory receptor CD28, and HLA-DR, an MHC class II cell surface receptor. As a co-inhibitory receptor, we focused on the expression of PD-L1.

For each molecule, two parameters were examined during the analysis: the percentage of cells expressing a given molecule (Fig 4B–4D, upper panel), and the geometric mean of molecule’s fluorescence intensity (Fig 4B–4D, lower panel). While the first indicates the abundance of cells in the culture, the later indicates the number of molecules expressed on the cells’ surface.

For the CD86 marker, analysis of samples incubated for 24h demonstrated that ~35% of monocytes were CD86 positive, and exposure to rhIFNγ increased the percentage to ~70% (Fig 4B, upper panel). Following 48 hours, the percentage of CD86 expressing monocytes was ~ 70%, and exposure to rhIFNγ had no additional effect (Fig 4B, upper panel). In terms of geometric mean, however, in samples incubated only with complete medium, similar cell surface amounts of molecules were detected following 24 and 48 hours, whereas exposure to rhIFNγ resulted in a dose-dependent elevation of CD86 expression at both time points (Fig 4B, lower panel).

For HLA-DR, almost 100% of monocytes expressed the molecule following 24 and 48 hours of incubation, regardless to rhIFNγ exposure (Fig 4C, upper panel). Analysis of geometric mean, however, demonstrated that the rhIFNγ led to substantial increase in HLA-DR cell-surface expression in a dose dependent manner, at both time points (Fig 4C, lower panel).

For PD-L1, dose-dependent effect of rhIFNγ was detected in terms of both cell percentage and geometric mean (Fig 4D). Interestingly, at 48 hours, we observed relatively high PD-L1 geometric mean values in some samples, even when the percentage of PD-L1^+^ cells remained modest (Fig 4D). This can occur when a small subset of monocytes appears to upregulate PD-L1 quite strongly, thereby inflating the average fluorescence intensity (geometric mean) even though most cells remain below the threshold for PD-L1 positivity. Consequently, a modest fraction of cells with very high PD-L1 expression can drive the geometric mean upward while leaving the percentage of positive cells relatively modest.

Additional experiments for exploring the kinetics of T cell and monocyte activation were conducted to improve the modeling of the MLR response (see Supporting Results and S3–S5 Figs).

### Immunophenotype feature extraction for immune response modeling

**Fig 5:**
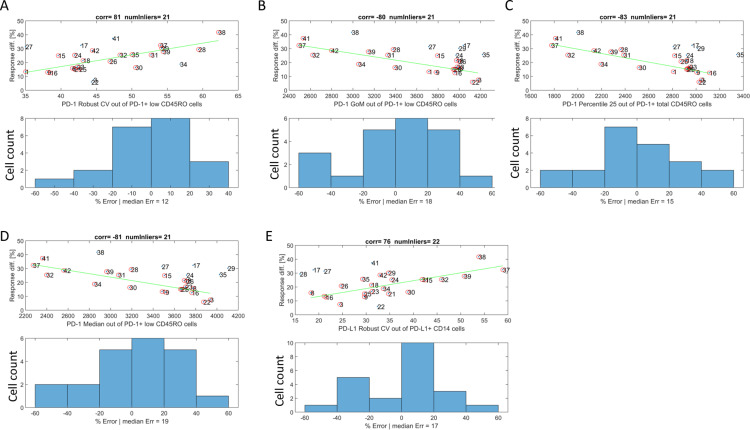
Correlation between T cell responsiveness and PD-1/PD-L1 expression parameters. For all panels, top graph is a linear fit, based on Random sample consensus (RANSAC) algorithm, and bottom graph is the distribution of errors (%) with respect to the estimator (green line). The correlation coefficient of the inliers and their number (out of 27 samples) are indicated at the top of each panel. Response diff [%] is the difference of T cells activation between samples activated with anti-CD3 + anti-PD-L1 (10^-8^M) and samples activated with anti-CD3 only. Labels of x-axes indicate the statistical parameters of PD-1 or PD-L1 distribution used for analysis.

Prior to the modeling process we assessed some of the basic immunophenotype information for their statistical prediction power. This imperative step is needed due to the large number of possible variables. Of note, multiple statistical measurements that describe the distributions of PD-1 and PD-L1 expression on cells were analyzed (e.g., mean, median, 25th percentile, Robust CV (coefficient of variation); Fig 5). For the baseline immunophenotyping, several measurements of PD-1 and PD-L1 expression levels were analyzed by flow cytometry, including the frequencies of T cells that express PD-1 and monocytes that express PD-L1, and the geometric mean of fluorescence intensity of PD-1 and PD-L1 (Fig 5). While frequency measurement (% cells) indicates the abundance of cells in the culture, the later (geometric mean) indicates the number of molecules expressed on the cells’ surface.

As described in Fig 2, the distribution of the different PD-1 and PD-L1 measures, of 27 individual blood samples, were analyzed to examine potential correlation between donors’ immunophenotype and T cells activation, in response to anti-PD-L1 treatment.

To identify the flow cytometry measures and statistics relevant for predicting the immune response in MLR experiments, the correlation of different baseline PD-1 and PD-L1 flow cytometry readouts and T cell activation intensities were examined.

Since the data contains relatively a significant number of outliers, we used the RANdom SAmple Consensus (RANSAC) algorithm to estimate the linear regression parameters (Fig 5A–5E, green line in the top panels) [26]. The correlation coefficient of the inliers and their number (out of 27 samples) are indicated at the top of each panel. Response diff [%] is the difference of T cells activation between samples activated with anti-CD3 + anti-PD-L1 (10^-8^M) and samples activated with anti-CD3 only. The analyses revealed that prediction of immune response, with error rate of less than 30% from values of ex vivo experiments (lower panel), were achieved for a broad range of fluorescence intensities covering the majority (80%) of samples.

As an initialization step in each Cell Studio simulation, the distribution of PD-1 expression over the T-cell agents was estimated using the geometric mean and the percentage of cells expressing PD-1 level above some threshold that were obtained from the FACS analysis. This distribution was utilized to predict the response of a particular blood sample.

### Performance of in silico vs. ex vivo MLR experiments

All the kinetic information from the biological experiments described above was used for model development and for the parameterization of the Cell Studio model, as described in the Material and Methods and in Fig 1.

**Fig 6:**
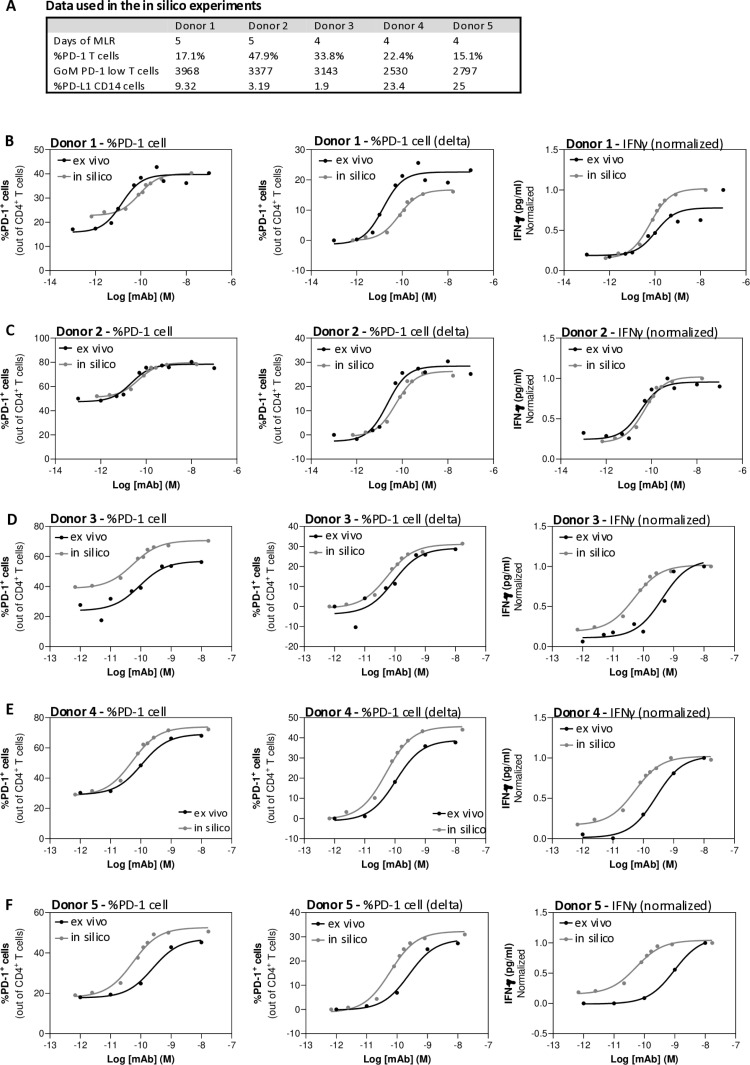
In silico vs. ex vivo MLR results demonstrating comparable dose response to anti-PD-L1 antibody. **(A)** Summary of the MLR parameters and baseline immunophenotype data used for simulations. **(B-F)** Curve fits of ex vivo and in silico results of individual donors. (B, C) 5 days MLR; (D-F) 4 days MLR. Left panel, results of %PD-1 T cells. Middle panel, results of delta %PD-1 T cells (the difference between %PD-1^+^ cells in samples treated with the indicated anti-PD-L1 concentrations minus the lowest concentration). Right panel, normalized IFNγ values.

To examine the performance of Cell Studio, results of in silico (virtual) vs. ex vivo (actual) MLR experiments were compared. PBMCs from five healthy donors were isolated, and their basic immunophenotype was analyzed at baseline, (prior to the MLR experiments). Fig 6A summarizes the MLR parameters, including number of days of MLR incubation, and PD-1/ PD-L1 immunophenotype data.

Experiments ran for a range of anti-PD-L1 concentrations, from 10^-12^M to 10^-7^M (0.00015–15 µg/ml), for 4 or 5 days, and results of T cell activation were compared (Fig 6B–6F). To allow accurate comparison of the anti-PD-L1 dose effect on the different T cell activation readouts, a sigmoid curve was fitted for each ex vivo and in silico experiment of each donor. The sigmoid function consists of 3 parameters that define its shape, including the Bottom, EC50 and Top. The Bottom parameter indicates the values of %PD-1^+^ T cells or IFNγ obtained with the lowest anti-PD-L1 concentration. The Top parameter indicates the values of %PD-1^+^ T cells or IFNγ obtained with the highest anti-PD-L1 concentration. The EC50 (half maximal effective concentration) is the concentration of anti-PD-L1 that results in 50% effect.

Using these sigmoid curves, anti-PD-L1 effect on T cell activation in the in silico setting was compared to that of the ex vivo experiments. Specifically, the percentages of PD-1 expressing CD4 T cells were compared; either ex vivo values (Fig 6, left panel) or delta values were used (i.e., the difference between %PD-1^+^ cells in samples treated with any anti-PD-L1 concentration and the lowest concentration; Fig 6, middle panel). For IFNγ, due to technical issues in determining absolute levels of the cytokine in ex vivo experiments, all results were normalized per experiment before comparing the results (Fig 6, right panel).

### Statistical comparison of in silico and ex vivo sigmoid curve parameters

**Fig 7:**
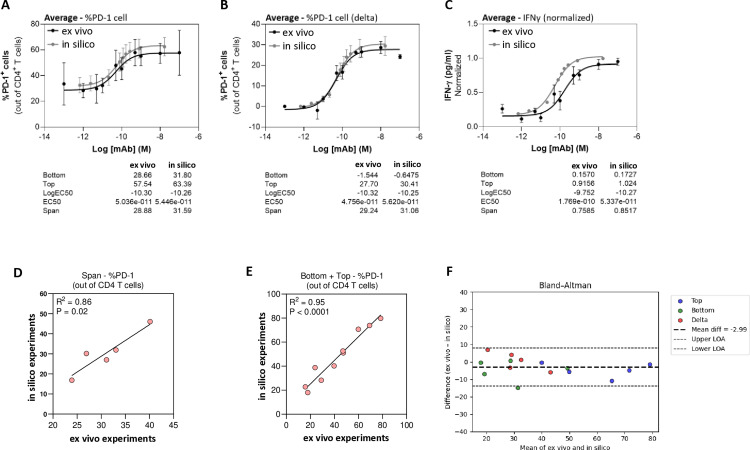
Comparison of in silico vs. ex vivo MLR results. **(A–C)** Average sigmoid curves of in silico vs. ex vivo results of all 5 donors. Below each graph, values of the Bottom, Top, and EC50 parameters of the sigmoid models are indicated. Span indicates the difference between the Top and Bottom parameters. **(A)** Results of %PD-1 T cells. **(B)** Results of delta %PD-1 T cells. **(C)** Normalized IFNγ values. **(D)** Correlation between in silico vs. ex vivo Span values of the dose response curve fits. **(E)** Correlation between in silico vs. ex vivo Bottom and Top parameter values of dose response curve fits. **(F)** Bland–Altman analysis of in silico vs. ex vivo results for the sigmoid parameters (Top, Bottom, and Delta). Each parameter set is color-coded (blue, green, and red, respectively). The combined mean difference (bias) is shown as a dashed black line, with 95% limits of agreement (±1.96 SD) represented by dashed lines. The majority of data points lie within these limits, indicating close agreement between in silico and ex vivo results and minimal systematic bias in the predictions.

To evaluate the performance of CellStudio, statistical analyses of the individual runs were conducted. In Fig 7, averaged sigmoid curves of all donors were compiled and the sigmoid parameters of in silico vs. ex vivo experiments for all activation readouts were evaluated, i.e., %PD-1 T cells (Fig 7A), delta %PD-1 T cells (Fig 7B), and normalized IFNγ (Fig 7C). High resemblance between the in silico and ex vivo PD-L1 dose response curves were observed.

To quantitatively measure the simulation performance, error and accuracy rates were calculated by Root Mean Squares (RMS). First, the RMS of Cell Studio results were calculated with respect to the sigmoid curves of ex vivo experiments, either ex vivo %PD-1 cells values or the delta values (S6A Fig). Analysis demonstrated average prediction accuracy of 74.3% with respect to the sigmoid curve of ex vivo (absolute) values, and 82.5% with respect to the sigmoid of the delta values.

To take into consideration the intrinsic variability of the ex vivo results, and to correct the prediction accuracy rates accordingly, the RMS and accuracy of ex vivo results, with respect to their own sigmoid, were also calculated. This analysis demonstrated average accuracy of 93.4% of ex vivo values with respect to the sigmoid curve of ex vivo (absolute) or delta values (S6B Fig).

To correct the prediction accuracy of Cell Studio, the %accuracy of Cell Studio predictions relative to the accuracy of ex vivo experiments was calculated. This analysis takes into consideration the variability of the ex vivo biological data, demonstrating average corrected prediction accuracy of 79.4% relative to the ex vivo absolute %PD-1 values, and 88.6% for the %PD-1 delta values (S6C Fig).

To further evaluate the performance of Cell Studio, paired t-test analysis was performed between the means of spans (the difference between the Top and Bottom parameters; S6D Fig), the top parameter (S6E Fig) and the bottom parameter (S6F Fig) of in silico vs. ex vivo experiments. No significant differences were detected in all comparisons.

Moreover, spans of %PD-1 T cells of in silico runs strongly correlated with those of ex vivo experiments (Fig 7D, R^2^ = 0.86, p = 0.02). Similar effects were also detected in correlation analysis of the Bottom + Top parameters, demonstrating highly accurate prediction capability of Cell Studio (Fig 7E, R^2^ = 0.95, p < 0.0001).

To further evaluate the agreement between predicted in silico and actual ex vivo measurements, we performed a Bland–Altman analysis on the three key parameters derived from the sigmoidal dose–response curves (“Top,” “Bottom,” and “Delta”). Fig 7F depicts a Bland–Altman plot in which each parameter set is color-coded and a combined mean difference (bias) ±1.96 SD is shown. The majority of data points lie within these limits of agreement, indicating close concordance between model predictions and experimental values, and the small mean difference implies minimal systematic over-estimation or under-estimation of the model.

Moreover, we conducted a benchmarking analysis to compare Cell Studio performance against established machine‑learning (ML) methods in predicting treatment effect on a held‑out test set using RMSE; Cell Studio outperformed the machine‑learning methods examined (S7 Fig and Supplementary Methods in S1 Text).

Collectively, these data indicate high performance level of the Cell Studio in predicting the effect of therapeutic antibody on T cells activation. Cell Studio can potentially be used as a viable tool for simulating complex immunological setups to assess the activation of T cells ex vivo and to predict the level of patient response to anti-PD-1/PD-L1 immunotherapy.

## Discussion

In the evolving landscape of cancer immunotherapy, particularly with immune checkpoint inhibitors, a significant proportion of patients remains non-responsive, highlighting a critical unmet need in personalized oncologic care [14]. Our study addresses this gap by utilizing ABM through the Cell Studio platform [18,19], a novel approach enabling predicting individual immune responses to immunotherapeutic antibodies. This method leverages the unique immunophenotype characteristics of each patient, offering a personalized predictive model of their response to anti-PD-L1 antibody treatment.

The strength of ABM lies in its ability to simulate complex biological systems with multiple interacting agents, such as cells in the immune system. Our application of this modeling approach in the context of immunotherapy allowed us to predict the ex vivo immune response of memory T cells to anti-PD-L1 blocking antibody with high precision. The strong correlation between the in silico predictions and ex vivo immune responses emphasizes the robustness of our model. Furthermore, the ability of the model in identifying responders and non-responders to immunotherapy underscores its potential clinical utility.

Our ABM predicts ex vivo immune activation with > 80% accuracy, whereas meta‑analyses examining established clinical biomarkers for anti‑PD‑1/PD‑L1 therapy, including PD‑L1 expression on tumor cells, tumor‑mutational burden (TMB), and tumor-infiltrating lymphocytes (TIL), report pooled AUCs of roughly 0.6–0.7 across tumor types [27]. Because those biomarkers are evaluated against clinical outcomes (RECIST response, PFS or OS) and our ABM is currently benchmarked against laboratory immune activation, the two performance metrics are not directly comparable. Nevertheless, the meta‑analysis results underscore the persistent challenge of accurately identifying ICI responders and point to an unmet need that complementary approaches could address. Our mechanistic, blood‑based model may help fill this gap, either as a stand‑alone assay or, more realistically, integrated into multimodal panels, once its immune‑activation scores are correlated with recognized clinical endpoints. Moving beyond mere predictive accuracy, the study offers deep insights into the biological mechanisms underpinning the immune response. Incorporation of biological parameters and cellular mechanisms driving differential responses in the Cell Studio simulation engine opens new avenues for the development of targeted immunotherapeutic strategies. These insights are particularly valuable in understanding the role of various cell types and signaling pathways, such as the PD-1/PD-L1 pathway, in modulating the immune response to immunotherapy.

A key element of the immune response modeled in our experiments is the baseline expression of PD-1 on T cells and PD-L1 on antigen-presenting cells (monocytes), which we find to be variable across individuals. Changes in the percentage of PD-1 expressing T cells or in their receptor density can affect the threshold for T cell activation, as these T cells are more susceptible to PD-1–mediated inhibition if not adequately blocked by anti-PD-L1 antibodies. Conversely, monocytes that express higher baseline levels of PD-L1 can drive more pronounced inhibitory interactions, dampening early T cell activation events. Once T cells begin to secrete IFNγ, PD-L1 can be further upregulated on monocytes, establishing a feedback loop that finely tunes immune activation levels.

In individuals with relatively lower PD-1 expression, T cells may require weaker PD-1/PD-L1 blockade to reach a robust activation state, whereas those with elevated PD-1 may need stronger or more sustained blockade to overcome inhibitory signals. Similarly, high PD-L1 expression on monocytes can prolong the inhibitory effect unless sufficient anti-PD-L1 antibody is present. These mechanistic insights into the interplay of PD-1 and PD-L1 expression suggest that baseline immunophenotypes, beyond simple markers of T-cell or monocyte abundance, can dictate distinct activation trajectories and clinical response patterns. Our agent-based model incorporates these variations through donor-specific immunophenotyping data, enabling a personalized simulation of T-cell–monocyte interactions and may explain how unique immunophenotypic profiles drive heterogeneity in patient responses to immunotherapy. This mechanistic precision underscores the potential utility of Cell Studio for optimizing therapeutic approaches that best align with the patient’s immune landscape.

A deeper examination of the model’s parameters also provides valuable mechanistic insights into how immunophenotype features shape response outcomes. By systematically adjusting or “knocking out” specific variables within the ABM (e.g., PD-1 expression thresholds, monocyte-to-T cell ratios, or PD-L1 induction kinetics), one can quantify each parameter’s influence on T cell activation and subsequent immune engagement. For instance, in our simulations, baseline PD-1 levels and monocyte PD-L1 expression emerged as primary determinants of whether a robust or reduced T cell response is achieved upon anti-PD-L1 blockade. Such analyses can further pinpoint why some individuals remain non-responsive, guiding more targeted therapeutic approaches and suggesting biomarker candidates for clinical stratification. In future work, correlating these in silico findings with a broader panel of immunophenotypic data (including additional checkpoints) could significantly refine predictive accuracy and deepen our understanding of the molecular events underlying response variability.

The clinical potential of incorporating models such as that of Cell Studio at the point-of-care settings is intriguing. By enabling the prediction of patient responses prior to treatment initiation, this approach offers a promising avenue for enhancing the precision of cancer therapy. It holds the potential to improve patient outcomes by personalizing treatment plans, reducing unnecessary risks associated with non-effective treatments, saving precious time and lowering healthcare costs. This paradigm shift towards personalized medicine in oncology underscores the transformative impact of predictive modeling in healthcare.

Considering future applications, the versatility of Cell Studio in adapting to emerging therapies, such as gene and cell therapies, further enhances its potential. The adaptability to model responses to a wide array of antibodies and cell therapies like chimeric antigen receptor (CAR)-T cells [28], CAR-Natural Killer (NK) cells [29,30], and TCR-engineered T cells [31], could position Cell Studio and similar frameworks as valuable tools in the ongoing evolution of personalized medicine.

Our methodology also demonstrates a robust approach for model design and parameterization using ex vivo experiment results. This approach, combing ex vivo/in silico workflow, offers a controlled method to derive biological insights, enhancing the accuracy and applicability of predictive modeling in biomedical research. The upcoming availability of the Cell Studio platform for academic and industry researchers may offer new opportunities for exploration and innovation in the field of immunotherapy.

Despite our focus on the PD-1/PD-L1 axis in this work, we recognize that other immunoregulatory pathways, such as TGF-β and CTLA-4, play significant roles in modulating tumor-immune dynamics. The absence of these additional checkpoints in our current model may limit its applicability to certain patient subgroups or tumor settings, although by focusing only on a minimal number of cellular pathways we avoid over-fitting. Furthermore, we do not yet incorporate additional immune cell populations such as regulatory T cells (Tregs) or B cells, nor do we account for potential cross-talk with pro- or anti-inflammatory cytokines beyond IFNγ. In addition, the model does not currently include key features of the tumor microenvironment, such as hypoxia and nutrient competition, which may lead to incomplete or inaccurate predictions in certain patient subgroups. Including these elements could improve our ability to capture complex immune interactions, particularly in tumors where non–PD-1/PD-L1 mechanisms predominate. Consequently, future versions of the Cell Studio platform will seek to incorporate these alternative pathways, providing a more holistic view of immune escape mechanisms and enhancing our ability to predict diverse therapeutic outcomes.

Looking ahead, several directions for future research could further advance the utility of our ABM approach. First, larger cohorts, drawn from diverse patient populations, will be essential to validate the model’s predictive accuracy and to ensure that it captures a broad range of immunophenotypic variations. Second, extending the model to other checkpoint inhibitors (e.g., anti-CTLA-4) or next-generation immunotherapies (e.g., bispecific antibodies, adoptive T-cell therapies) would provide insights into its generalizability and potentially uncover additional mechanistic features. Third, incorporating high-dimensional immune profiling technologies, such as single-cell transcriptomics or multiparameter flow cytometry, may enhance the model’s granularity and predictive power by providing richer immunophenotypic data. Ultimately, such expansions could facilitate the integration of ABM-based methods into clinical decision-making, enabling more personalized and effective treatments across a wider spectrum of oncologic and immunologic conditions.

From a clinical perspective, several steps would be required to translate our ex vivo predictions into actionable patient care. First, prospective validation in larger, disease-specific cohorts is needed to confirm the reproducibility and accuracy of the ABM under varied clinical contexts. Second, integrating streamlined assays for high-throughput sample processing, such as microfluidic isolation of immune cells and rapid immunophenotyping, could enable a more feasible, point-of-care workflow. Third, bridging studies that compare ex vivo readouts to in vivo endpoints (e.g., tumor regression, survival) will be critical to reinforce clinical utility and identify any gaps in the current model. Finally, although our data suggest a strong correlation between ex vivo and simulated results, it is important to acknowledge that in vivo complexity, including tissue-specific microenvironments and additional immunosuppressive pathways, may attenuate or amplify responses observed ex vivo. Addressing these factors through iterative modeling and validation will be essential for the successful clinical adoption of this approach.

Future validation of our ABM approach in more complex in vivo settings can include patient-derived xenograft or humanized mouse models. These systems will enable us to capture additional layers of tumor-immune complexity, such as spatial heterogeneity, metabolic constraints, and interactions with other stromal components. By comparing the ABM’s predictions to longitudinal outcomes in these in vivo models, we can further refine the model’s parameters and improve its clinical relevance. Such validation efforts will also provide an opportunity to include additional immunoregulatory pathways, thereby enhancing the model’s predictive accuracy and facilitating its integration into personalized immunotherapy workflows.

Additionally, this study’s validation was performed on a small cohort of donors, all of whom were healthy rather than cancer patients. While this design allowed us to develop a controlled ex vivo system, it may not capture the full spectrum of immune dysfunction or tumor-specific factors that occur in oncology settings. Moreover, the MLR assay does not replicate the complexities of an in vivo tumor microenvironment, including local immunosuppressive signals and spatial heterogeneity. In addition, because our ABM is calibrated through user-defined parameters and assumptions derived from ex vivo data, there is a risk of algorithmic bias if these parameters do not account for the immunological diversity in different patient populations. Addressing these limitations, by expanding sample sizes, incorporating patient-derived samples, and refining the ex vivo model to better capture in vivo conditions will be essential to enhance the clinical relevance of our findings.

Complementing these analyses, we benchmarked our ABM approach against several standard ML regression models on a held-out test set. Despite the very limited training cohort (22 donors), the ABM outperformed all examined ML methods, underscoring its resilience under data-scarce conditions, an advantage in most healthcare applications. This superior performance likely reflects the ABM’s incorporation of mechanistic, biology-driven rules that guide predictions even when sample sizes are small, whereas purely data-driven ML models typically require much larger datasets to avoid overfitting.

In conclusion, our study using an integrative approach of basic immunophenotyping and ABM has successfully demonstrated the ability to simulate and predict T cell responses in MLR experiments. The ability to predict patient responses to immune checkpoint inhibitors may represent a significant advancement in the field of personalized medicine, paving the way for more effective and individualized cancer treatments.

## Supporting information

S1 Fig Flow cytometry gating strategy for measuring the distribution of peripheral blood T cell subpopulations. CM: central memory T cells; EM: effector memory T cells.
(PDF)

S2 Fig %CD14^+^ monocytes throughout MLR kinetics. The percentage of CD14^+^ cells in MLR cultures were examined by flow cytometry in (A) activated samples or (B) non-activated samples, following 24h, 48h, 72h and 96h of incubation. Non-activated samples include T-cells:monocytes co-cultures incubated in complete medium without anti-CD3 or anti-PD-L1 (No Abs), with IgG isotype control, or with different anti-PD-L1 concentrations (10^-8^-10^-12^M). Activated samples were all incubated with anti-CD3, and with different concentrations of the anti-PD-L1 antibody or IgG control. Data is shown as mean ± s.e.m.
(PDF)

S3 Fig IFNγ effect on CD45RO T cells viability and activation, as determined by inhibitory molecules expression level. (A-C). rhIFNγ (0.02ng-20ng) was added to isolated CD45RO positive cells incubated in 0.2 ml complete medium (concentration range of 0.1–100ng/ml) for 4h and 24h. (A) % cells in “live” gate; (B) %PD-1 high expressing cells; (C) %KLRG1 expressing cells. Upper panel demonstrates % cells and lower panel demonstrates geometric mean of PD-1 and KLRG1, respectively. Data is shown as mean ± s.e.m.
(PDF)

S4 Fig Early IFNγ secretion by memory T cells and PD-L1 expression by monocytes 4 hours following incubation. (A) IFNγ levels in the medium after 2h and 4h of MLR incubation were measured using ELISA. (B) %PD-L1 expressing CD14 cells following 1h, 2h or 4h of MLR were determined by flow cytometry. Data is shown as mean ± s.e.m. n.a – no antibody.
(PDF)

S5 Fig IFNγ stability and consumption by monocytes. Different rhIFNγ concentrations (250–1000pg/ml) were added to complete medium and incubated at 37^o^C for 24 or 48h. IFNγ at T0 indicates the concentration in samples that were not incubated. IFNγ levels were measured using ELISA.
(PDF)

S6 Fig Evaluation of Cell-Studio performance in predicting anti-PD-L1 dose effect on T cells activation. **(A) Error and accuracy rates of Cell-Studio prediction (uncorrected).** Error rates of prediction (in the original data units) were calculated by RMS of Cell-Studio results at all simulated anti-PD-L1 concentrations with respect to the sigmoid curves of actual ex vivo experiments. %RMS was calculated by ratio of RMS with the span of corresponding sigmoid curve. % Accuracy was calculated as 100% minus %RMS. **(B) Error and accuracy rates between actual experiment results and their sigmoid estimator**. Error rates (in data units) was calculated by RMS of actual results with respect to their own sigmoid curves. %RMS was calculated by ratio of RMS with the span of corresponding sigmoid curve. % Accuracy was calculated as 100% minus %RMS. **(C) Cell-Studio %accuracy relative to accuracy of actual experiments.** %accuracy was calculated using the following equation: %cellStudio accuracy/ % actual experiments accuracy * 100. **(D) Comparison of actual (ex vivo) vs. virtual (in silico) span (Top minus Bottom parameters) of dose response curve fits.** Paired t-test between the spans of %PD-1 T cells generated in the actual and virtual experiments. **(E) Comparison of actual (ex vivo) vs. virtual (in silico) Bottom parameter of dose response curve fits.** Paired t-test between the Bottom values of %PD-1 T cells generated in the actual and virtual experiments. **(F) Comparison of actual (ex vivo) vs. virtual (in silico) Top parameter of dose response curve fits.** Paired t-test between the Top values of %PD-1 T cells generated in the actual and virtual experiments.
(PDF)

S7 Fig Comparing predictive performance of Cell Studio vs. six machine learning regression models. Bars show the root mean squared error (RMSE) in predicting the per-donor change in percentage of PD-1–expressing CD4 T cells (%ΔPD-1 CD4 cells) across five held-out test donors. The ML models used for the analysis included Linear Regression, Ridge, Lasso, Random Forest, Support Vector Regression (SVR), and XGBoost. All models were trained on 22 donors using three immunophenotype features (%PD-1 T cells; GoM PD-1 low T cells; %PD-L1 CD14 cells), with hyperparameters tuned via grid search and leave-one-out cross-validation (LOOCV). Lower RMSE indicates closer agreement between predicted and observed treatment effects.
(PDF)

S1 Text Supporting information text including supplementary results and material and methods. (DOCX)

S1 Data Supporting information including raw data used for analyses. (ZIP)
