# Supplementary figures and images for: Agent-based modeling for personalized prediction of an experimental immune response to immunotherapeutic antibodies
Source: PLoS One. 2025 Jun 9;20(6):e0324618. doi: 10.1371/journal.pone.0324618 (PMC12148075; doi:10.1371/journal.pone.0324618)

# Supplementary Figure 1

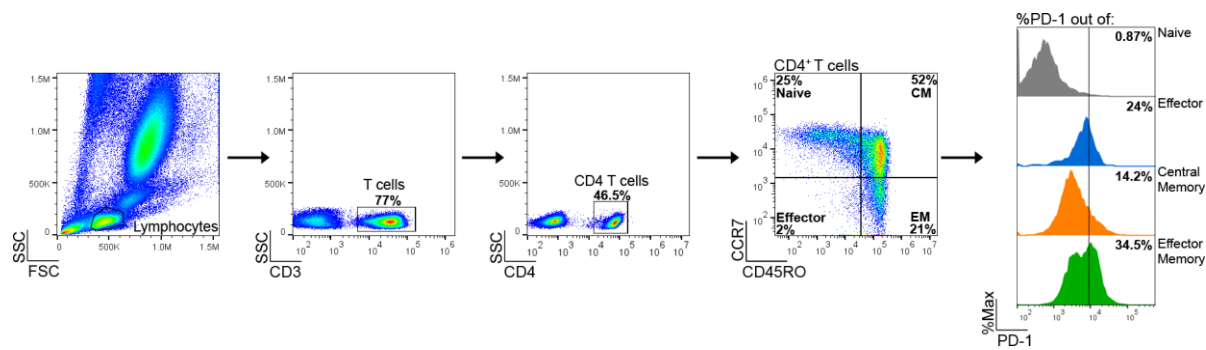

Supplement: S1 Fig — CM: central memory T cells; EM: effector memory T cells. (PDF) [file pone.0324618.s001.pdf]

# Supplementary Figure 2

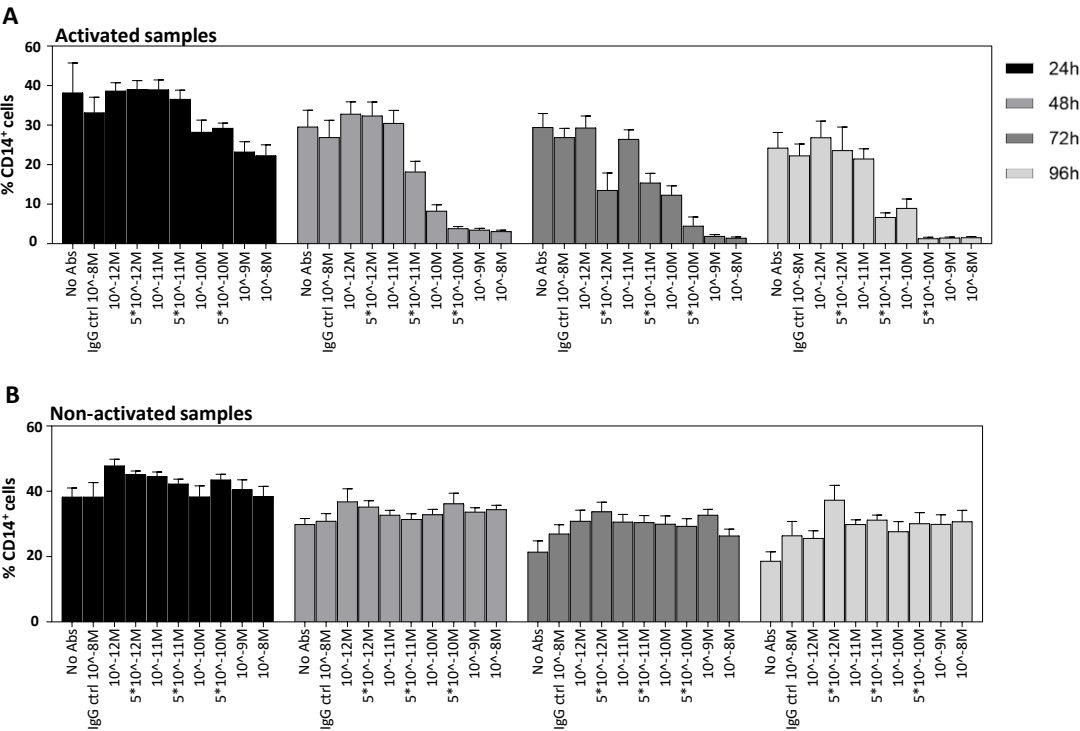

Supplement: S2 Fig — The percentage of CD14+ cells in MLR cultures were examined by flow cytometry in (A) activated samples or (B) non-activated samples, following 24h, 48h, 72h and 96h of incubation. Non-activated samples include T-cells:monocytes co-cultures incubated in complete medium without anti-CD3 or anti-PD-L1 (No Abs), with IgG isotype control, or with different anti-PD-L1 concentrations (10-8-10-12M). Activated samples were all incubated with anti-CD3, and with different concentrations of the anti-PD-L1 antibody or IgG control. Data is shown as mean ± s.e.m. (PDF) [file pone.0324618.s002.pdf]

# Supplementary Figure 3

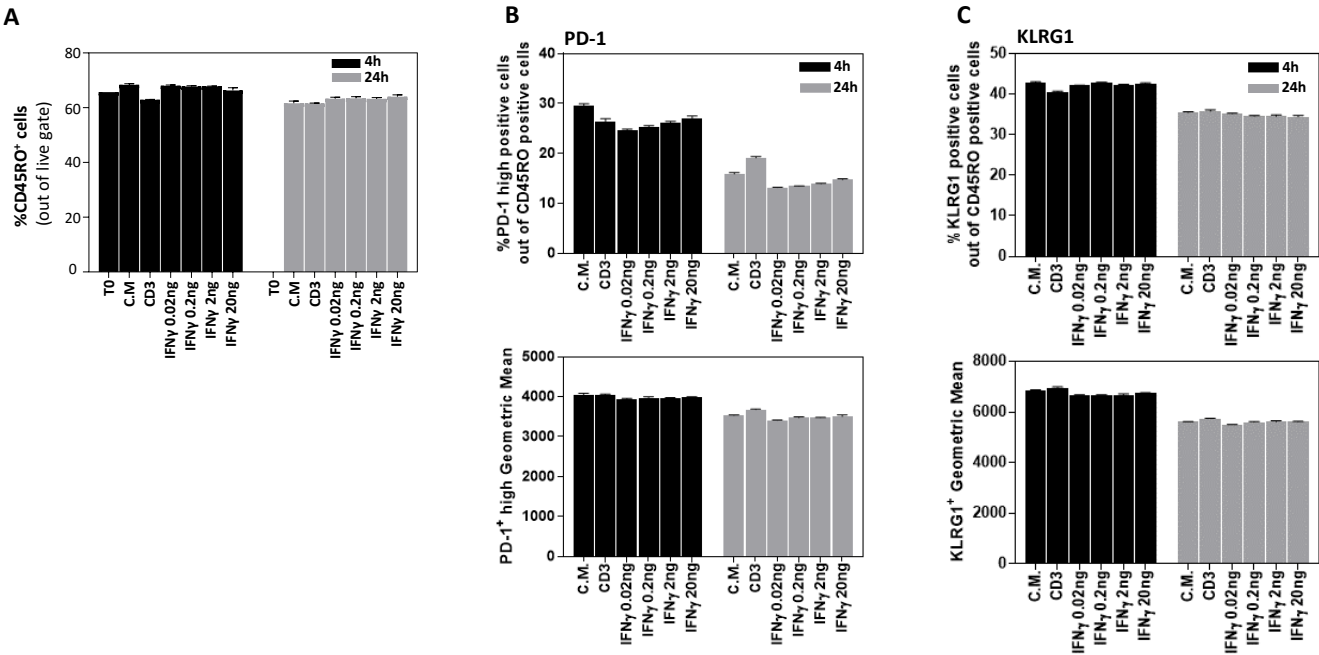

Supplement: S3 Fig — (A-C). rhIFNγ (0.02ng-20ng) was added to isolated CD45RO positive cells incubated in 0.2 ml complete medium (concentration range of 0.1–100ng/ml) for 4h and 24h. (A) % cells in “live” gate; (B) %PD-1 high expressing cells; (C) %KLRG1 expressing cells. Upper panel demonstrates % cells and lower panel demonstrates geometric mean of PD-1 and KLRG1, respectively. Data is shown as mean ± s.e.m. (PDF) [file pone.0324618.s003.pdf]

# Supplementary Figure 4

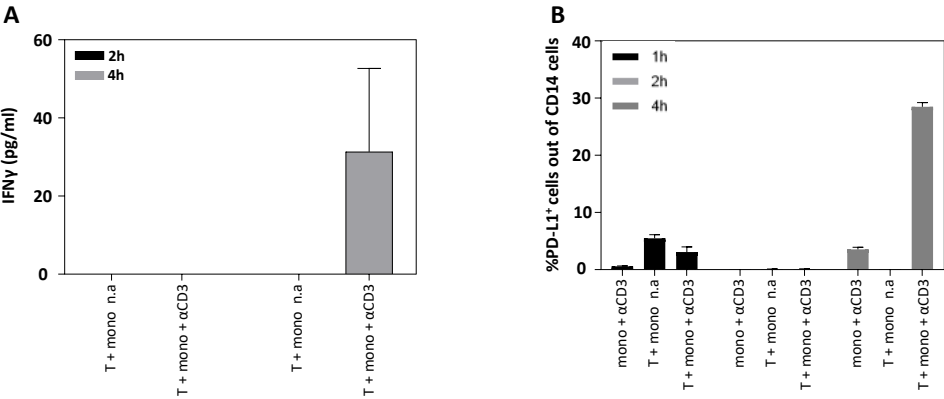

Supplement: S4 Fig — (A) IFNγ levels in the medium after 2h and 4h of MLR incubation were measured using ELISA. (B) %PD-L1 expressing CD14 cells following 1h, 2h or 4h of MLR were determined by flow cytometry. Data is shown as mean ± s.e.m. n.a – no antibody. (PDF) [file pone.0324618.s004.pdf]

# Supplementary Figure 7

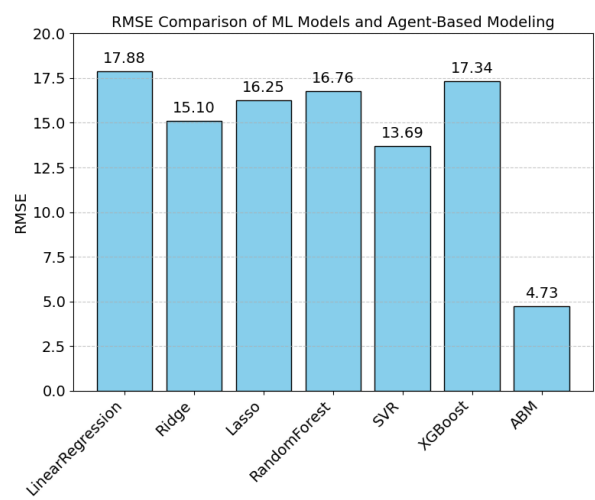

Supplement: S7 Fig — Bars show the root mean squared error (RMSE) in predicting the per-donor change in percentage of PD-1–expressing CD4 T cells (%ΔPD-1 CD4 cells) across five held-out test donors. The ML models used for the analysis included Linear Regression, Ridge, Lasso, Random Forest, Support Vector Regression (SVR), and XGBoost. All models were trained on 22 donors using three immunophenotype features (%PD-1 T cells; GoM PD-1 low T cells; %PD-L1 CD14 cells), with hyperparameters tuned via grid search and leave-one-out cross-validation (LOOCV). Lower RMSE indicates closer agreement between predicted and observed treatment effects. (PDF) [file pone.0324618.s007.pdf]
