# Supplementary material for: Agent-based modeling for personalized prediction of an experimental immune response to immunotherapeutic antibodies
Source: PLoS One. 2025 Jun 9;20(6):e0324618. doi: 10.1371/journal.pone.0324618 (PMC12148075; doi:10.1371/journal.pone.0324618)
Supplement: S5 Fig — Different rhIFNγ concentrations (250–1000pg/ml) were added to complete medium and incubated at 37oC for 24 or 48h. IFNγ at T0 indicates the concentration in samples that were not incubated. IFNγ levels were measured using ELISA. (PDF) [file pone.0324618.s005.pdf]

Supplementary Figure 5

**Medium**	**Activator**	**IFNγ at T_0** (pg/ml)	**IFNγ 24h** (pg/ml)	**IFNγ at 48h** (pg/ml)
Complete medium	IFNγ	1000	17	17
	IFNγ	500	3.5	9.8
	IFNγ	250	0	4.2
Complete medium + monocytes	-	0	0	0
	Anti-CD3	0	0	0
	IFNγ	1000	41.3	25.7
	IFNγ	500	16.1	12.1
	IFNγ	250	6.3	2.6
