# Supplementary material for: Agent-based modeling for personalized prediction of an experimental immune response to immunotherapeutic antibodies
Source: PLoS One. 2025 Jun 9;20(6):e0324618. doi: 10.1371/journal.pone.0324618 (PMC12148075; doi:10.1371/journal.pone.0324618)

Supplementary Figure 6

A

		Donor 1	Donor 2	Donor 3	Donor 4	Donor 5	Average
%PD-1 absolute values	RMS (in data units)	3.7	2.4	16.5	7.4	10.8	8.2
	%RMS	15.5	7.7	50.0	18.4	36.9	25.7
	%Accuracy	84.5	92.3	50.0	81.6	63.1	**74.3**
%PD-1 delta values	RMS (in data units)	7.6	1.4	2.4	11.6	4.0	5.4
	% RMS	31.8	4.5	7.3	28.9	14.9	17.5
	% Accuracy	68.2	95.5	92.7	71.1	85.1	**82.5**

B

		Donor 1	Donor 2	Donor 3	Donor 4	Donor 5	Average
%PD-1 absolute values	RMS (in data units)	2.1	2.3	3.7	0.8	1.1	2.0
	%RMS	8.6	7.4	11.1	2.0	3.7	6.6
	%Accuracy	91.4	92.6	88.9	98.0	96.3	**93.4**
%PD-1 delta values	RMS (in data units)	2.0	2.3	3.7	0.8	1.1	2.0
	% RMS	8.5	7.5	11.2	2.0	3.7	6.6
	% Accuracy	91.5	92.5	88.8	98.0	96.3	**93.4**

C

% CellStudio accuracy relative to accuracy of "wet" experiments						
	Donor 1	Donor 2	Donor 3	Donor 4	Donor 5	Average
%PD-1 absolute values	92.5	99.6	56.3	83.3	65.5	**79.4**
%PD-1 delta values	74.6	103.2	104.4	72.6	88.4	**88.6**

D

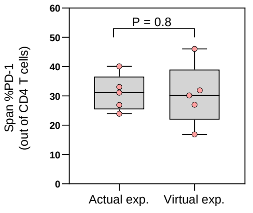

E

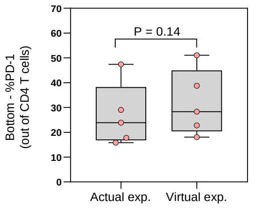

F

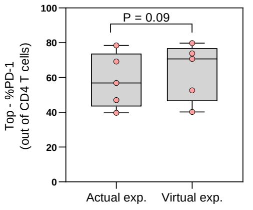

Supplement: S6 Fig — (A) Error and accuracy rates of Cell-Studio prediction (uncorrected). Error rates of prediction (in the original data units) were calculated by RMS of Cell-Studio results at all simulated anti-PD-L1 concentrations with respect to the sigmoid curves of actual ex vivo experiments. %RMS was calculated by ratio of RMS with the span of corresponding sigmoid curve. % Accuracy was calculated as 100% minus %RMS. (B) Error and accuracy rates between actual experiment results and their sigmoid estimator. Error rates (in data units) was calculated by RMS of actual results with respect to their own sigmoid curves. %RMS was calculated by ratio of RMS with the span of corresponding sigmoid curve. % Accuracy was calculated as 100% minus %RMS. (C) Cell-Studio %accuracy relative to accuracy of actual experiments. %accuracy was calculated using the following equation: %cellStudio accuracy/ % actual experiments accuracy * 100. (D) Comparison of actual (ex vivo) vs. virtual (in silico) span (Top minus Bottom parameters) of dose response curve fits. Paired t-test between the spans of %PD-1 T cells generated in the actual and virtual experiments. (E) Comparison of actual (ex vivo) vs. virtual (in silico) Bottom parameter of dose response curve fits. Paired t-test between the Bottom values of %PD-1 T cells generated in the actual and virtual experiments. (F) Comparison of actual (ex vivo) vs. virtual (in silico) Top parameter of dose response curve fits. Paired t-test between the Top values of %PD-1 T cells generated in the actual and virtual experiments. (PDF) [file pone.0324618.s006.pdf]
