# Supplementary material for: Agent-based modeling for personalized prediction of an experimental immune response to immunotherapeutic antibodies
Source: PLoS One. 2025 Jun 9;20(6):e0324618. doi: 10.1371/journal.pone.0324618 (PMC12148075; doi:10.1371/journal.pone.0324618)
Supplement: S1 Text — (DOCX) [file pone.0324618.s008.docx]

­­­

Supporting information

Agent-Based Modeling for Personalized Prediction of an Experimental Immune Response to Immunotherapeutic Antibodies

Omri Matalon^1^, Andrea Perissinotto^2^, Kuti Baruch^1^, Shai Braiman^1^, Anat Geiger Maor^1^, Eti Yoles^1^, Ella Wilczynski^2^, Uri Nevo^2,3^, Avner Priel^*, 2,4^

^1^ ImmunoBrain, Ltd., 3 Haim Pekeris St., Rehovot 7670203, Israel.

^2^ Department of Biomedical Engineering, The Iby and Aladar Fleischman Faculty of Engineering, Tel Aviv University, Tel Aviv 6997801, Israel.

^3^ Sagol School of Neuroscience, Tel Aviv University, Tel Aviv 6997801, Israel.

^4^ Faculty of Engineering, Ruppin Academic Center, Emek Hefer 4025000, Israel

* Corresponding Author

# Supplementary Materials & Methods­­

IFNγ ELISA

Following incubation period of MLR experiments, supernatants were collected and IFN-γ was measured by ELISA (Biolegend; Human IFN-γ ELISA MAX™ Deluxe, cat. #430105) according to manufacturer’s instructions.

Flow cytometry

Cells were incubated with FcR Blocking Reagent (Miltenyi Biotech; cat. #130-059-901), to prevent non-specific binding of the mAbs to Fc receptors. Multicolor flow cytometry analysis was performed to assess surface-marker expression of T cells and monocytes using the following mAbs, purchased from Biolegend: FITC anti-human CD8 (Cat. 344704), PE anti-human PD-L1 (Cat. 329706), PerCP-Cy5.5 anti-human CD14 (Cat. 325622), APC anti-human PD-1 Cat. 329908, APC/Cy7 anti-human CD4 (Cat. 317418). Cells were stained for 30 min in ice cold FACS buffer (PBS,2% FBS, 5mM EDTA). Data were acquired on a CytoFLEX Flow Cytometer (Beckman Coulter) and analyzed using FlowJo 10.2 (Tree Star, Ashland, OR, USA).

For the baseline basic immunophenotyping, several measurements of PD-1 and PD-L1 expression levels were analyzed by flow cytometry, including the frequencies of T cells that express PD-1 and monocytes that express PD-L1, and the geometric mean of fluorescence intensity of PD-1 and PD-L1. While the first measurement (% cells) indicates the abundance of cells in the culture, the later (geometric mean) indicates the number of molecules expressed on the cells’ surface. Of note, multiple statistical measurements that describe the distributions of PD-1 and PD-L1 expression on cells (e.g. mean, median, 25th percentile, Robust CV) were analyzed and used for the modelling process.

Prior to the validation experiments using Cell Studio, the distribution of the different PD-1 and PD-L1 measures, of the individual blood samples, were analyzed to examine potential correlation with T cells activation, in response to anti-PD-L1 treatment.

PD-L1 receptor occupancy

PD-L1 receptor occupancy (RO) was assessed by Flow cytometry for modeling the binding of anti-PD-L1 antibody to its ligand. PBMCs were thawed, counted and 1x10^5^ cells per sample were seeded in a 96U-shape bottom wells. Cells were centrifuge, incubated with FcR Blocking Reagent (Milteny, cat#130-059-901) for 10 minutes at 4^o^c, and washed with FACS buffer (2% FBS, 5mM EDTA in PBS). The plate was then centrifuged, and cell pellets were incubated for 30 minutes at 4^o^c with decreasing concentration of indicated anti-PD-L1 antibody, ranging from 1*10^-7^M to 6.5*10^-13^M. In parallel, equivalent samples were incubated with similar concentrations (1*10^-7^M - 6.5*10^-13^M) of isotype control antibody to measure background signal. Samples were analyzed in duplicates or triplicates.

Cells that were incubated with 1*10^-7^M of antibody were used to check the maximal PD-L1 occupancy and referred as “anti-PD-L1 saturated” samples. The lower antibody concentrations were used to measure the dose dependent PD-L1 receptor occupancy over T cells, and referred as “tested” samples.

Following incubation with anti-PD-L1, cells were washed 3 times with FACS buffer and stained with FITC anti-human IgG-Fd region secondary antibody (Thermo, cat# SA5-10189) and APC/Cy7 anti-CD3 antibody (Biolegend, cat#300318) for 30 minutes at 4^o^c. Cells were then washed 3 times and staining for FITC anti-human IgG-Fd was assessed by flow cytometry. %PD-L1 RO on pre-gated lymphocytes population (CD3^+^ cells) was assessed for each sample according to the following calculation:

| %RO = $\frac{GM(tested sample)-background GM(corresponding IgG control sample)}{GM(saturated sample)-background GM(corresponding IgG control sample)} X 100$ |  |
| --- | --- |

Where GM refers to the geometric mean, the corresponding IgG control sample refers to cells that were incubated with hIgG1 isotype control antibody at concentration similar to that of anti-PD-L1 antibody of a specific “tested” sample.

%RO of each sample was calculated as average of replicates. Negative values after subtraction of background signal were considered as 0% RO. RO results were used for modeling the binding of anti-PD-L1 antibody to its ligand.

**Antibodies**

For PD-L1 blocking assays, a hIgG1 anti-PD-L1 antibody, produced by AkesoBio, was used. For controls, a hIgG1 isotype control antibody was used, also produced by AkesoBio.

## Governing Equations of the CellStudio Simulations

T-Cell mathematical description

The set of variables describing a T cell in the model are as follows:

1. $\boldsymbol{F}_{\boldsymbol{aCD}\boldsymbol{3}}$ is the stimulatory effect of anti-CD3 binding. It is supposed that the initial concentration of anti-CD3 is enough to bind all the CD3 receptor on T-Cell, raising the stimulatory strength that can be achieved via the CD3 pathway to its maximum value, thus in all the simulations the following was set:

| $\boldsymbol{F}_{\boldsymbol{aCD}\boldsymbol{3}}\text{=}1$ |  |
| --- | --- |

[Eq S1]

1. $\boldsymbol{F}_{\boldsymbol{CoSt}}$ is the co-stimulatory effect of the CD28 binding. The contribution of the co-stimulatory receptors is given by:

| $\boldsymbol{F}_{\boldsymbol{CoSt}}\text{ =}f\left( {CD28}_{Ex},{CD80}_{Ex} \right)= \frac{\left( \frac{{CD80}_{Ex}}{{CD28}_{Ex}} \right)^{0.5}}{kF2+\left( \frac{{CD80}_{Ex}}{{CD28}_{Ex}} \right)^{0.5}} \in[0,1]$ |  |
| --- | --- |

[Eq S2]

where $\boldsymbol{kF}\boldsymbol{2}$ represent the slope of the modified Hill function, ${\boldsymbol{CD}\boldsymbol{28}}_{\boldsymbol{Ex}}$ is the percentage of expressed CD28 on T-Cell’s membrane and ${\boldsymbol{CD}\boldsymbol{80}}_{\boldsymbol{Ex}}$ is the percentage of expressed CD80 on Monocyte’s membrane. The Hill function here is used to describe the relation between the receptor-to-ligand ratio and their effect on the stimulation of the Tcell, in a function that rises from zero and reaches the maximal level in a sigmoidal shape. This method is employed since, as explained earlier, receptor levels are measured relatively, hence, we express their level in the range (0,1).

1. $\boldsymbol{F}_{\boldsymbol{Inh}}$ is the inhibitory effect of PD-1 binding to PD-L1. It is defined by:

| $\boldsymbol{F}_{\boldsymbol{Inh}}\text{ = }f\left( {PD1}_{ex},{PDL1}_{Bind} \right)=\frac{\left( \frac{{PD1}_{Ex}}{{PDL1}_{Bind}} \right)^{0.5}+{0.8*PD1}_{Ex}}{kF1+\left( \frac{{PD1}_{Ex}}{{PDL1}_{Bind}} \right)^{0.5}}+e^{-kExpF_{Inh}*{PD1}_{Ex}}$ |  |
| --- | --- |

[Eq S3]

Where $\boldsymbol{kF}\boldsymbol{1}$ is the slope of the modified Hill function, ${\boldsymbol{PD}\boldsymbol{1}}_{\boldsymbol{Ex}}$ is the percentage of expressed PD-1 on T-Cell’s membrane and ${\boldsymbol{PDL}\boldsymbol{1}}_{\boldsymbol{Bind}}$ is the percentage of Monocyte’s PD-L1 occupied by anti-PD-L1. The first term is obtained from a Hill function in which the numerator is given more weight to the ${PD1}_{Ex}$, hence the T-Cell is more sensitive to this property than to${PDL1}_{Bind}$. The second term (exponential) which highly contributes to the inhibition only for very low values of ${PD1}_{Ex}$ as experimental data shows.

Taking into account these contributions, the overall activation of the T-Cell is given in Eqs S4-5 below (also described in the Material & Methods section):

| $A_{coeff}=-k_{Inh}\text{*}\boldsymbol{F}_{\boldsymbol{Inh}}+(1+k_{Cost}*\boldsymbol{F}_{\boldsymbol{CoSt}}\text{)}*(k_{aCD3}*\boldsymbol{F}_{\boldsymbol{aCD}\boldsymbol{3}})$  [Eq S4] |  |
| --- | --- |

where $\boldsymbol{F}_{\boldsymbol{Inh}}$, $\boldsymbol{F}_{\boldsymbol{CoSt}}$, $\boldsymbol{F}_{\boldsymbol{aCD}\boldsymbol{3}}$ are the functions of the three processes described in Eqns. [S1]-[S3] and $\boldsymbol{k}_{\boldsymbol{Inh}}$**,** $\boldsymbol{k}_{\boldsymbol{Cost}}$, $\boldsymbol{k}_{\boldsymbol{aCD}\boldsymbol{3}}$ are the weights given to these three processes.

| $\boldsymbol{Act}\boldsymbol{=}\frac{\left( 1+\tanh\left( \beta\cdot\left( A_{coeff}-\theta\right) \right) \right)}{2}+Inhib_{general}$  $\frac{d(Inhib_{general})}{dt}=-k_{general}*(1-Inhib_{general\text{ }}))$ |  |
| --- | --- |

[Eq S5]

where $\boldsymbol{\beta}$ and $\boldsymbol{\theta}$ are parameters that define the slope and the position of the curve and $\boldsymbol{Inhi}\boldsymbol{b}_{\boldsymbol{general}}$ takes into account, collectively, the other inhibitory mechanisms.

From experimental data it is found that the activation of T-Cell determines the increase of ${PD1}_{Ex}$ on the cell surface and the release of IFNγ to the medium.

The expression of PD-1 (${\boldsymbol{PD}\boldsymbol{1}}_{\boldsymbol{Ex}}$) is a process that start inside the cell with the production of PD-1 receptor. These receptors are initially accumulated inside the cell and after a certain amount of time expressed on the surface. The model of this behavior is realized using two differential equations:

| $\frac{{dPD1}_{in}}{dt}={(k}_{PDIn\_Act}*(0.1+Act))*(1-{PD1}_{ex})-(k_{PD1In\_Ex}*{PD1}_{in}))$  $\frac{{dPD1}_{ex}}{dt}=k_{PD1In\_Ex}*{PD1}_{in}$ |  |
| --- | --- |

[Eq S6]

Where ${\boldsymbol{PD}\boldsymbol{1}}_{\boldsymbol{in}}$ is the percentage of PD-1 accumulated inside the cell, ${\boldsymbol{PD}\boldsymbol{1}}_{\boldsymbol{ex}}$ is the percentage of expressed PD-1 on T-Cell’s membrane, $\boldsymbol{k}_{\boldsymbol{PDIn\_Act}}$ is the weight coefficient of the influx of activation on ${PD1}_{in}$, and $\boldsymbol{k}_{\boldsymbol{PD}\boldsymbol{1}\boldsymbol{In\_Ex}}$ is the coefficient that sets the fraction of the PD-1 produced exposed on the membrane.

The production of ${PD1}_{in}$ is reduced if its quantity grows and also while ${PD1}_{ex}$ approach the maximum expression. The same concept applies for IFNγ which is produced inside the cell and after some time is being released.

| $\frac{{dIFN}_{in}}{dt}={(k}_{IFNIn\_Act}*(0.1+Act))*(MaxIFN-{IFN}_{ex})-(k_{IFNIn\_Ex}*{IFN}_{in}))$  $\frac{{d(IFN}_{ex})}{dt}=k_{IFNIn\_Ex}*{IFN}_{in}$  [Eq S7] |  |
| --- | --- |

Where $\boldsymbol{IFN}_{\boldsymbol{in}}$ denotes the number of IFNγ molecules accumulated inside the T-cell, $\boldsymbol{IFN}_{\boldsymbol{ex}}$is the number of IFNγ molecules released by the T-Cell, $\boldsymbol{k}_{\boldsymbol{IFNIn\_Act}}$ is the weight coefficient of the IFNγ production activation**,** $\boldsymbol{k}_{\boldsymbol{IFNIn\_Ex}}$ is the fraction of IFNγ released to the medium. The constant $MaxIFN$ = 15000 has been evaluated experimentally.

### Monocyte mathematical description

The model of Monocyte differentiates from the T-Cell model due to the absence of an activation variable. The expression of the two receptors (PD-L1 and CD80) depends on the concentration of IFNγ sensed by the Monocyte. It is defined by a function of the IFNγ concentration that is mapped into a normalized value:

| $\boldsymbol{F}_{\boldsymbol{IFN}}\text{ }\text{=}f\left( IFN\gamma\right)=\frac{\left[ IFN\gamma\right]}{kF3+\left[ IFN\gamma\right]}\in\left[ 0 1 \right]$  [Eq S8] |  |
| --- | --- |

The production and expression of PD-L1 and CD80 follows the same model shown for PD-1 in T-Cell in Eq S6: Initially the receptors are produced in the cell and after a predefined duration, they are being expressed on the surface. The equations describing PD-L1 and CD80 dynamics appear below:

| $\frac{{dPDL1}_{in}}{dt}=k_{PDLIn}\text{* }\boldsymbol{F}_{\boldsymbol{IFN}}*(1-{PDL1}_{ex})-(k_{PDL1\_Ex}*{PDL1}_{in}))$  $\frac{{dPDL1}_{ex}}{dt}=k_{PDL1\_Ex}*{PDL1}_{in}$ |  |
| --- | --- |

[Eq S9]

where ${\boldsymbol{PDL}\boldsymbol{1}}_{\boldsymbol{in}}$ is the percentage of PD-L1 accumulated inside the Monocyte, ${\boldsymbol{PDL}\boldsymbol{1}}_{\boldsymbol{ex}}$ is the percentage of PD-L1 expressed on Monocyte’s membrane, $\boldsymbol{k}_{\boldsymbol{PDLIn}}$ is the weight coefficient of $IFN\gamma$ on PD-L1 production**,** $\boldsymbol{k}_{\boldsymbol{PDL}\boldsymbol{1\_Ex}}$ is the weight coefficient of the PD-L1 fraction exposed on the membrane.

| $\frac{{dCD80}_{in}}{dt}=k_{CD80In}*\boldsymbol{F}_{\boldsymbol{IFN}}*(1-{CD80}_{ex})-(k_{CD80\_Ex}*{CD80}_{in}))$  $\frac{{dCD80}_{ex}}{dt}=k_{CD80\_Ex}*{CD80}_{in}$  [Eq S10] |  |
| --- | --- |

where ${\boldsymbol{CD}\boldsymbol{80}}_{\boldsymbol{in}}$ is the percentage of CD80 accumulated inside the Monocyte, ${\boldsymbol{CD}\boldsymbol{80}}_{\boldsymbol{ex}}$ is the percentage of CD80 expressed on Monocyte’s membrane, $\boldsymbol{k}_{\boldsymbol{CD}\boldsymbol{80}\boldsymbol{In}}$ is the weight coefficient influx of $IFN\gamma$ influx on CD80 production**,** $\boldsymbol{k}_{\boldsymbol{CD}\boldsymbol{80\_Ex}}$ is the weight coefficient of the CD80 fraction exposed on the membrane.

**Benchmarking Cell Studio vs. Machine Learning Approaches**

We evaluated the performance of Cell Studio against six standard machine learning (ML) regression models in predicting the treatment effect, defined as the per‐donor change in the percentage of CD4 T cells expressing PD‑1 between the highest and lowest anti‑PD‑L1 concentrations. The ML models include Linear Regression, Ridge, Lasso, Random Forest, SVR and XGBoost. Data from 22 donors were used, with the predicted outcome value computed as (%PD‑1‑expressing CD4 at the highest anti-PD-L1 concentration) minus (%PD‑1‑expressing CD4 at the lowest anti-PD-L1 concentration). Three immunophenotype variables were used as features, including %PD‑1 T cells, GoM PD‑1 low T cells, and %PD‑L1 CD14 cells. Intermediate concentrations were not modeled to avoid overfitting given the limited sample size. For each ML method we built a pipeline of hyperparameter tuning over a wide range: Ridge/Lasso α = 0.01–100; Random Forest n_estimators = 50–200, max_depth = None, 3, 5, 10; SVR C = 0.1–10 with linear and rbf kernels; XGBoost n_estimators = 50–200, max_depth = 3–7, learning_rate = 0.01–0.2. Hyperparameters were selected via leave‑one‑out cross‐validation (LOOCV), which maximizes training data usage and provides nearly unbiased error estimates on small datasets. All ML models were retrained on the full 22‑donor cohort using their optimal settings, and then used to predict a held‑out test set of five donors. Final performance was assessed by root mean squared error (RMSE) score. The results are displayed as bar graphs.

# Supplementary Results

## Exploring the kinetics of monocyte death throughout the MLR

In previous MLR experiments that were assessed over 5 days incubation timeframe, a major reduction in the percentage of monocytes was detected, indicating on cell death throughout the process (data not shown). Importantly, this reduction was specifically detected in samples that underwent cellular activation, suggesting that T cells stimulation led to monocyte cytotoxicity, either directly or indirectly.

Since the abundance of monocytes over time is essential for accurate modeling of the MLR settings, the percentage of CD14 positive cells was analyzed in samples of the MLR kinetic experiments. Flow cytometry data demonstrated a gradual reduction in the percentage of monocytes over time following cellular activation with both anti-CD3 and high concentrations of anti-PD-L1 (above 1x10^-11^M) (**S2A Fig**), as compared to non-activated cells (**S2B Fig**)***.*** These results suggest that monocyte death is a consequence of T cells activation, *in-vitro*.

Several mechanisms can be responsible for the reduction in monocyte numbers, including the presence of high anti-PD-L1 levels in the medium, IFNγ induced monocytes activation that leads to apoptosis, or activity of CD8 cytotoxic T lymphocytes (CTL). To examine the effect of anti-PD-L1 concentration on this process, T cell-monocyte cultures were incubated with increasing doses of anti-PD-L1, but without anti-CD3 to prevent T cells activation. No reduction in monocyte percentage was detected in the presence of all anti-PD-L1 antibody concentrations, relative to cells incubated with IgG isotype control or without anti-PD-L1 antibody (**S2B Fig**). These data indicate that monocyte death is not induced by the anti-PD-L1 antibody per se, but rather, as a result of T cell activation that occurs only following strong stimulation with anti-CD3 combined with PD-1/PD-L1 blockade.

## Exploring the effects of IFNγ on T cells viability and cellular activation.

The effects of IFNγ on T cells viability and activation were examined as well. To this end, CD45RO^+^ memory T cells were isolated from PBMCs of healthy donors and incubated in either complete medium without additives, with anti-CD3 antibody, or with increasing concentration of rhIFNγ. Flow cytometry analysis revealed no changes in the abundance of memory T cells in the culture, regardless to rhIFNγ concentration, and relatively to the negative control. These results suggest that the secreted IFNγ throughout the MLR assay does not affect T cells viability (**S3A Fig**).

To determine IFNγ effect on T cell activation, the percent of cells expressing PD-1 or KLRG1 (additional immune checkpoint expressed on activated T cells), and their geometric means, were monitored at early time points, following 4 and 24 hours of incubation. Flow cytometry analysis demonstrated that exposure of memory T cells to rhIFNγ did not affect PD-1 and KLRG1 expression, in terms of both cell abundance and amounts of molecules on the cell surface, relative the controls (**S3B** and **S3C Figs**, upper and lower panels, respectively). These results indicate that IFNγ does not induce T cell activation, and therefore direct influence of IFNγ on T cell stimulation was omitted from Cell Studio equations defining the T cell state machine.

## Exploring early activation of T cells and monocytes

To model the kinetics of T cell and monocyte activation at early time points, MLR experiments of 1-4 hours of incubation were conducted. These experiments were used to determine the time point when IFNγ accumulation in the medium and PD-L1 upregulation on monocytes begin. This is critical for the correct modeling of the kinetics of cellular activation and for achieving accurate prediction in the in silico experiments. For that end, T cells and monocytes were co-cultured for 1, 2, or 4 hours in the presence of anti-CD3 antibody, to induce T cell activation. As negative controls, cells were incubated without the anti-CD3 antibody, or monocytes were incubated with anti-CD3 in the absence of T cells. IFNγ and elevation in PD-L1 expressing monocytes were detected only in activated samples and following 4 hours of incubation, as compared to the negative controls (**S4A and S4B Figs**). Importantly, these results are in agreement with the literature data regarding the duration of T-monocytes immunological synapse formation (i.e., 4 hours). This suggests that T cell and monocyte activation start following 4 hours of incubation, after forming at least one stable immunological synapse and accomplishing a stable interaction. These data were incorporated in the Cell Studio model.

## Exploring IFNγ stability in culture conditions

During the MLR response, IFNγ is being continuously produced and secreted. Our initial assumption was that a portion of the molecules is consumed by the monocytes, This should result in a reduction of IFNγ levels that should be observed at late time points. An alternative mechanism for this reduction might involve IFNγ degradation due to short half-life in culture conditions. To address these issues, rhIFNγ was incubated at different concentrations (ranging from 0-1000pg/ml) in complete medium at 37^o^_C_ for 24 and 48 hours, in the presence or absence of isolated monocytes. Concentration of IFNγ in the medium was then measured using ELISA. Strikingly, we found that more than 95% of the incubated IFNγ was degraded following 24 hours, relative to samples that were not incubated at 37 ^o^_C_ (IFNγ at T_0_; **S5A Fig, upper panel**). In addition, the presence of monocytes had no additional effect of the reduction of IFNγ levels (**S5A Fig, lower panel**). These results suggest that IFNγ is constantly degraded during the period of MLR incubation, a phenomenon that was essential to incorporate into the modelling process.
